# Protein Nanospheres and Nanofibers Prepared by Ice-Templating for the Controlled Release of Hydrophobic Drugs

**DOI:** 10.1021/acsanm.4c03657

**Published:** 2024-09-13

**Authors:** Meina Zhang, Hong Cai, Haifei Zhang

**Affiliations:** Department of Chemistry, University of Liverpool, Crown Street, Liverpool L69 7ZD, U.K.

**Keywords:** ice templating, protein scaffolds, nanofibers, microspheres, drug delivery

## Abstract

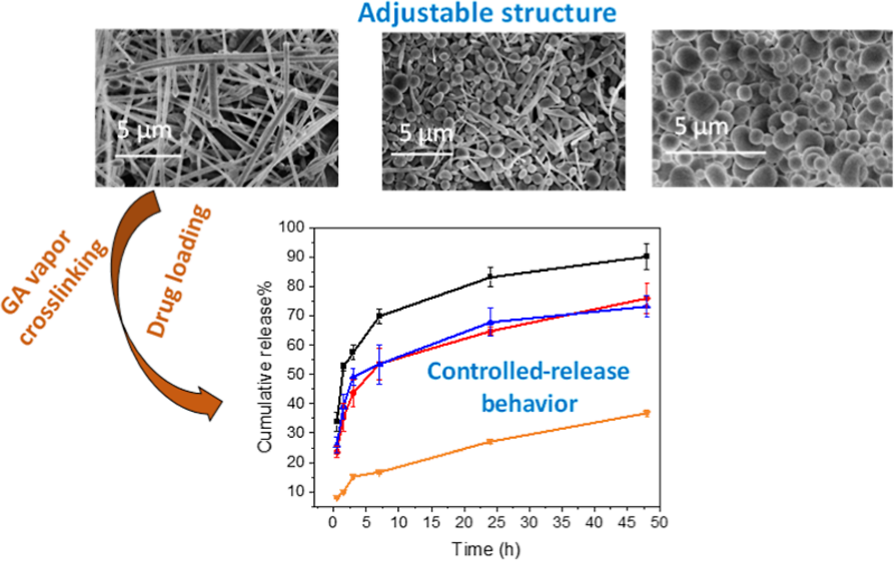

Protein scaffolds play a vital role in drug delivery systems. However, few research studies have been focused on loading hydrophobic drugs on protein scaffolds in biomedical fields. Here, we report on the development of protein microspheres and nanofibers by a simple ice-templating approach and their use as scaffolds for the controlled release of hydrophobic drugs, with bovine serum albumin (BSA) as the model protein and curcumin as the model hydrophobic drug. The BSA scaffolds display the unique nanofibrous and microspherical structures. This is a surprising discovery because there has been no report on the formation of microspheres via simple ice-templating of solutions or suspensions. To further understand the formation of microspheres by this approach, lysozyme, papain, and their composites with BSA are also studied. It is speculated that nanoparticles are first formed in aqueous BSA solution, attributed to the overlapping of hydration layers and autoassembly of inner hydrophobic cores of BSA globular molecules. Nanoprecipitation and soaking evaporation approaches are then used to load curcumin into the BSA scaffolds, followed by cross-linking with glutaraldehyde vapor to improve stability in an aqueous medium. The controlled release of curcumin is demonstrated, paving the way for various hydrophobic drugs loaded into this biodegradable and nonimmunogenic protein scaffold for potential treatments of diverse diseases.

## 1 Introduction

In the request for the release of active pharmaceutical ingredients and desired therapy, drug delivery systems have become an increasingly prevalent researching area in pharmaceutical and biomedical fields.^1^ Among diverse drug delivery systems, protein scaffolds are widely employed to deliver drugs, genes, and cells owing to the high loading capacity, ability to extend the delivery of drugs at a controlled time frame, potential to prevent infection upon surgery, directing cell activities, nontoxicity, as well as nonimmunogenicity.^2,3^ To date, various protein scaffolds have been developed for drug delivery, for instance, serving as bone repairing composite materials based on silk protein–protein interfacial bonding,^4^ regulating DNA condensation by a histone-like nucleoid structuring protein scaffold,^5^ culturing 3T3 fibroblasts and cells on a three-dimensional network scaffold of lysozyme,^6^ as well as binding to inactive split intein/enzyme extein protein fragments for splicing and activation of the luciferase enzyme.^7^ However, most research focused on the application of protein scaffolds in controlling cell attachment and proliferation, delivering genes, or regulating enzyme activities. Few studies have investigated the loading of hydrophobic pharmaceutical ingredients on protein scaffolds for the treatment of chronic or persistent medical conditions and diseases.^8^

Serum albumins are the most abundant proteins (52–62%) in blood plasma, acting as a carrier for transporting numerous endogenous or exogenous substances, fatty acids, ions, as well as small molecules.^9^ Compared to other serum albumins, bovine serum albumin (BSA) has been used and investigated widely due to its excellent stability, wide availability, low cost, and similar structures (76%) to human serum albumin.^10,11^ Two major binding sites (site I and site II) of BSA provide a versatile platform for encapsulating small hydrophobic drugs and bioactive ingredients within protein networks.^12^ With the merits of avoiding systemic clearance and degradation via natural mechanisms, nontoxicity, biocompatibility, and biodegradability, BSA becomes a more powerful candidate than synthetic polymers and plays an important role in modulating hydrophobic drug delivery in biological environments.^13^ The common method to prepare BSA nanofibrous scaffolds is electrospinning, where it is necessary to blend BSA with different polymers or surfactants and choose suitable solvents with desired solution viscosity, surface tension, and solution conductivity.^14,15^ Nevertheless, natural conformations of BSA structures are destroyed to some extent due to the involvement of denaturing agents and organic solvents. Additionally, coelectrospinning with synthetic polymers more or less deteriorates prevailing biocompatibility and immunogenicity of BSA. It is thus crucial to develop BSA nanofibrous scaffolds via a more versatile approach where no disadvantageous reagents, detrimental solvents, and less biocompatible materials are used.

Ice templating is an effective approach to fabricate nanofibers and only aqueous solutions or suspensions are employed without any unfavorable additions, indicating that no structural damage to materials may occur during the preparation process.^16^ Motivated by the development of BSA/polymer nanofibers by ice templating in our previous study, we are interested in the formation of pure BSA nanofibers via the same method.^17^ It was noticed that some microspheres were formed at a higher mass ratio of BSA and poly(vinyl alcohol) (PVA).^17^ This was a surprising finding because polymer microspheres are usually formed via the use of emulsions/dispersions or spraying (where the droplets act as templates).^18−22^ The use of polymer microspheres for drug loading and release has been employed extensively.^23−26^ We are not aware of polymer or ceramic microspheres produced by ice templating (that is, freezing a bulk solution and followed by freeze-drying, excluding the spray freezing technique).^16,27,28^ The production of polymer and protein microspheres via ice templating would provide a simple yet highly effective approach.

**Scheme 1:**
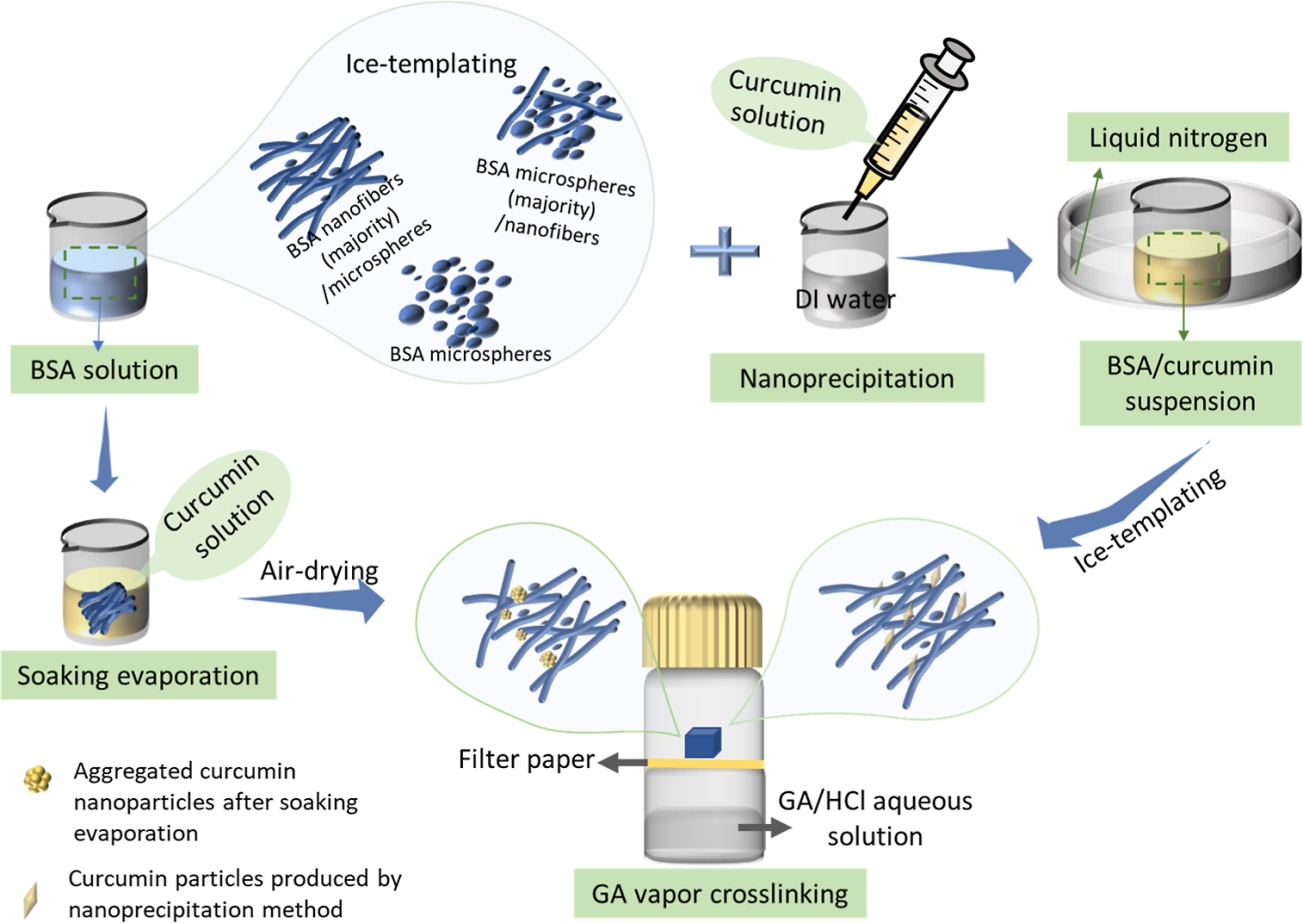
Schematic Illustration of Preparation of BSA Scaffolds with Adjustable Structures of Nanofibers and Microspheres via Ice-Templating of Aqueous BSA Solution Two types of loading approaches (nanoprecipitation and solvent evaporation) are used to upload curcumin into BSA scaffolds, which are subsequently crosslinked by glutaraldehyde (GA) vapor to increase stability.

Herein, we report the construction of BSA scaffolds with the structures of nanofibers and microspheres via a simple ice-templating approach, which were then uploaded with a hydrophobic drug (curcumin). As illustrated in Scheme 1, BSA scaffolds were composed of nanofibers and microspheres, the amount of which could be adjusted by varying concentrations of BSA solutions and mixing with other proteins, such as lysozyme. Nanoprecipitation and soaking evaporation methods were employed to load curcumin into the BSA scaffolds. Owing to the unstable issue of BSA scaffolds in a wet environment, BSA/curcumin composites were further cross-linked by glutaraldehyde (GA) vapor. Curcumin release behaviors could be effectively varied by changing the curcumin loading methods, which opens the way for the loading and release of other hydrophobic drugs in this biodegradable and nonimmunogenic protein scaffold.

## 2 Experimental Section

### 2.1 Chemicals and Materials

BSA (≥96%), curcumin from Curcuma longs (Turmeric), lysozyme from chicken egg white power (70,000 U/mg), papain from *Carica papaya* power (≥3 U/mg), chitosan (medium molecular weight between 190 and 310 kDa), sodium hydroxide (≥98%), phosphate-buffered saline (PBS) tablets (pH 7.2–7.6, 1 tablet/200 mL water), poly(vinyl alcohol) (89–98 kDa, 99+% hydrolyzed), and Bradford reagent (for 0.1–1.4 mg/mL protein) were purchased from Sigma-Aldrich. Ethanol and acetone were supplied by Fisher Scientific. Glutaraldehyde (GA, 50% aqueous solution) and hydrochloric acid (37%) were provided by Alfa Aesar. Acetic acid (100%) was provided by BDH. All chemicals were of analytical grade and used without further treatment.

### 2.2 Preparation of BSA Nanofibers/Nanospheres under Different Freezing Conditions and pH

Different amounts of BSA were dissolved in deionized (DI) water while continuously stirring at room temperature to form aqueous solutions at the concentrations of 0.5 1.5, 3, 5, and 10 mg/mL. The BSA solutions were subsequently frozen at different temperatures, i.e., in liquid nitrogen, freezer (−20 °C), or dry ice/acetone. To prepare the dry ice/acetone cold bath, dry ice was slowly added into acetone until no bubbles were formed and the temperature reached −78 °C. The frozen samples were then subjected to a freeze-drying process for 48 h using a Cool Safe freeze-dryer. In order to vary the pH of the BSA solutions (1.5 mg/mL), hydrochloric acid and sodium hydroxide were added in DI water, respectively, producing acidic and basic aqueous medium at pH 2 and pH 10 at room temperature. After BSA was added, the resulting acidic and basic BSA solutions were frozen in liquid nitrogen and then freeze-dried for 48 h.

### 2.3 Preparation of Porous Lysozyme, Papain, and Their Composites with BSA

Lysozyme and papain were, respectively, dissolved in DI water to produce the solutions at the concentrations of 1.5, 3, and 5 mg/mL at room temperature (20 °C). Additionally, the BSA solution (1.5 mg/mL) was mixed with lysozyme or papain solutions (1.5 mg/mL) at different volume ratios of 1:1, 1:2, and 2:1. These solutions were frozen in liquid nitrogen and freeze-dried for 48 h.

### 2.4 Curcumin Loading on BSA Nanofibers/Microspheres and Subsequent GA Vapor Cross-Linking

Two approaches (nanoprecipitation and solvent evaporation) with four different procedures were used to load curcumin on BSA nanofibers/microspheres scaffolds. The first and second procedures were nanoprecipitation with and without surfactant. Typically, 3 mL of curcumin/ethanol (3 mg/mL) solution was injected dropwise into 10 mL of DI water in the presence or absence of Tween 80 (0.5 v/v %) at a rate of 1 mL/min, followed by continuous stirring for 24 h to remove ethanol. The resulting suspension was centrifugated at 5000 rpm for 5 min. The orange precipitates produced were mixed with 10 mL of BSA aqueous solution (1.5 mg/mL) and subsequently stirred for 10 min to distribute curcumin precipitates evenly in the BSA solution. The mixture was frozen in liquid nitrogen and subsequently subjected to a freeze-drying process for 48 h.

The third procedure was nanoprecipitation without surfactant and subsequent centrifugation. 1.5 mL of curcumin/ethanol (3 mg/mL) solution was injected into 5 mL of DI water. The volume was reduced to 2.5 mL after continuous stirring for 48 h. In order to keep the same concentration of BSA solutions as those in first and second methods, 2.5 mL of DI water and 5 mL of BSA solution (3 mg/mL) were added. The mixture was stirred for 10 min and then frozen in liquid nitrogen and freeze-dried for 48 h. The fourth loading method was soaking evaporation. Around 20 mg of BSA material (prepared from 1.5 mg/mL BSA solution) was soaked in 4 mL of curcumin/ethanol (3 mg/mL) for 72 h, until all ethanol had evaporated.

The curcumin loading efficiency was determined by UV–vis analysis. 5 mg Cur-BSA composites were placed in 100 mL of PBS/ethanol solution (volume ratio 1:1) for 24 h until all samples were dissolved. According to the calibration curve of curcumin in PBS/ethanol solution (volume ratio 1:1) (Figure S1a), the absorbance of the yellow solution was determined as curcumin at 432 nm, and the amount in the PBS/ethanol solution was considered as loaded curcumin.
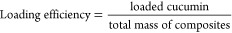


Cur-BSA composites produced by different approaches were placed in a sealed glass vessel, containing 6 mL of GA (50% aqueous solution), 6 mL of DI water, and 1 mL of hydrochloric acid (37%).^17^ With the treatment of GA vapor cross-linking for 24 h at room temperature, the resulting composites were placed in a vacuum oven overnight at room temperature to remove the unreacted GA.

### 2.5 Curcumin Release and Stability of BSA Scaffolds

Different release media were used for the curcumin release study. (i) 3.0 mg of GA-Cur-BSA composite was put in 30 mL of PBS/ethanol solution (volume ratio 1:1). 100 μL of PBS/ethanol (100 μL) was withdrawn at different time intervals and placed in a 96-well plate for UV–vis test at 432 nm. The withdrawn 100 μL solution was then added back to the PBS/ethanol release medium. (ii) 6.8 mg of GA-Cur-BSA composite was placed in 30 mL of PBS. 100 μL of PBS solution (100 μL) was withdrawn at the predetermined time intervals. After measuring the absorbance at 427 nm, the withdrawn 100 μL was put back into the release medium.

The stability of the BSA scaffolds was also evaluated by assessing the release of BSA into the medium. 15.0 mg of GA-Cur-BSA composite was placed in 20 mL of PBS at room temperature. 50 μL of PBS was withdrawn at different time intervals and mixed with 1.5 mL of Bradford reagent. After 6 min, 0.1 mL of this mixture was placed in a 96-well plate for the UV–vis test at 596 nm. The amount of dissolved BSA in PBS was determined based on the absorbance and the calibration curve (Figure S1b).

### 2.6 Characterization

The morphology of the freeze-dried materials was determined by scanning electron microscopy (SEM) (Hitachi S4800 SEM). Samples were placed onto double-sided adhesive carbon tape and then coated by gold at a sputter current of 15 mA for 45 s. A Vertex 70 Fourier transform infrared (FT-IR) spectrometer was used to detect and monitor the change of chemical groups at the wavenumber range of 600–4500 cm^–1^. Thermogravimetric analysis (TGA) (NETZSCH TG 209 F1 Libra) was conducted at a heating rate of 25 °C/min to 900 °C in nitrogen. BSA materials prepared under different pH conditions were inspected by a TA differential scanning calorimeter (DSC) programmed for a heat–cool–heat method at a heating rate of 10 °C/min to 150 °C, a cooling rate of 10 °C/min to −80 °C, and isothermal for 1 min. Zeta potential and particle size of protein and polymer solutions were characterized by dynamic light scattering (DLS) from the Malvern Instruments Ltd. with water as the dispersant at 25 °C. Element microanalysis was carried out on GA-cross-linked materials using a Thermo Scientific FlashSmart CHN Elemental Analyzer. The release of curcumin and BSA were determined using a Thermoscientific Varioskan Lux Plate Reader at 25 °C. Crystallinity of curcumin particles produced by different methods was explored by powder X-ray diffraction (PXRD) on a Bruker-AXS D8 ADVANCED diffractometer with Co radiation (Kα1 = 1.789010 Å) source in reflection geometry.

## 3 Results and Discussion

### 3.1 Formation of BSA Nanofibers/Microspheres via Ice-Templating

**Figure 1:**
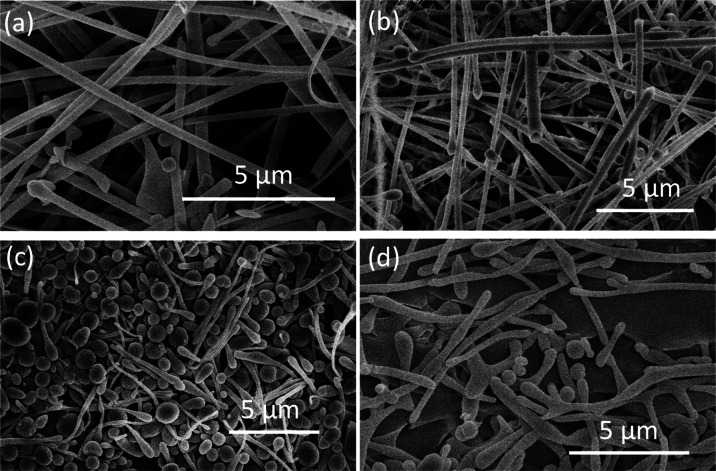
Morphology of freeze-dried BSA from aqueous BSA solutions frozen in liquid nitrogen at different concentrations: (a) 0.5, (b) 1.5, (c) 3.0, and (d) 5.0 mg/mL.

We observed that the morphology of BSA-PVA composites changed from nanofibers to the presence of microspheres when a higher ratio of BSA to PVA (3:1) was employed.^17^ It is known that porous materials can be produced by ice templating and polymer nanofibers are formed when a very dilute polymer solution is freeze-dried.^16^ However, to the best of our knowledge, there is no report on the formation of polymer microspheres when a simple ice-templating approach is employed, i.e., without combining ice templating with spraying or emulsion templating. Therefore, BSA nanofibers are expected to be formed (Figure 1a,b) when the dilute aqueous BSA solutions (concentrations 0.5 and 1.5 mg/mL, respectively) were frozen in liquid nitrogen and freeze-dried. The fiber diameters are around 400 nm. A close look shows spherical ends to some fibers. Surprisingly, when the BSA concentration was increased to 3 mg/mL, the majority of the structure exhibited the presence of microspheres (around 1–2 μm) with some short nanofibers (Figure 1c). When the BSA concentration was increased further to 5 mg/mL, larger lumps and less amount of shorter fibers was produced (Figure 1d). Further concentration increase to 10 mg/mL produced flakes of materials, with no fibers or microspheres (Figure S2). Therefore, this study was then focused on BSA concentrations up to 5 mg/mL.

Because freezing temperature has considerable impact on the morphology of ice-templated materials, we investigated if the formation of nanofibers and particularly microspheres could be observed when BSA solutions were frozen in dry ice/acetone (−78 °C) and a freezer (−20 °C). For the BSA samples prepared by freezing in dry ice/acetone, larger quantities of nanofibers were formed for the BSA concentrations of 0.5 and 1.5 mg/mL (Figure S3a,b). This is similar to freezing in liquid nitrogen. However, when the BSA concentration was increased to 3 mg/mL, unlike freezing in liquid nitrogen, only a small percentage of the structures was microspheres (Figure S3c). The structure change was significant when the BSA solutions were frozen in a freezer (−20 °C). No nanofibers or nanospheres were produced, and only large lumps were formed (Figure S4).

These results demonstrate the obvious impact of the freezing temperature on the morphology of ice-templated BSA materials. This may be explained by the varied temperature gradient and freezing rate. Temperature in the freezer (−20 °C) was higher than the homogeneous nucleation temperature of water, that is, around −48.1 °C.^29^ In this case, the ice nucleation rate was slower than the equilibrium rate of liquid, indicating that BSA molecules diffusion still took place toward the ice–water interface and other places simultaneously at −20 °C.^30^ With slow growth of ice crystals in the freezer, BSA molecules moved together, leading to a lump structure after the removal of ice crystals in the freeze-drying process. With very limited mobility at −196 °C (liquid nitrogen) and −78.5 °C (dry ice/acetone), BSA molecules could be excluded by growth of ice crystals, producing BSA nanofibrous and spherical structure after the freeze-drying process.

**Figure 2:**
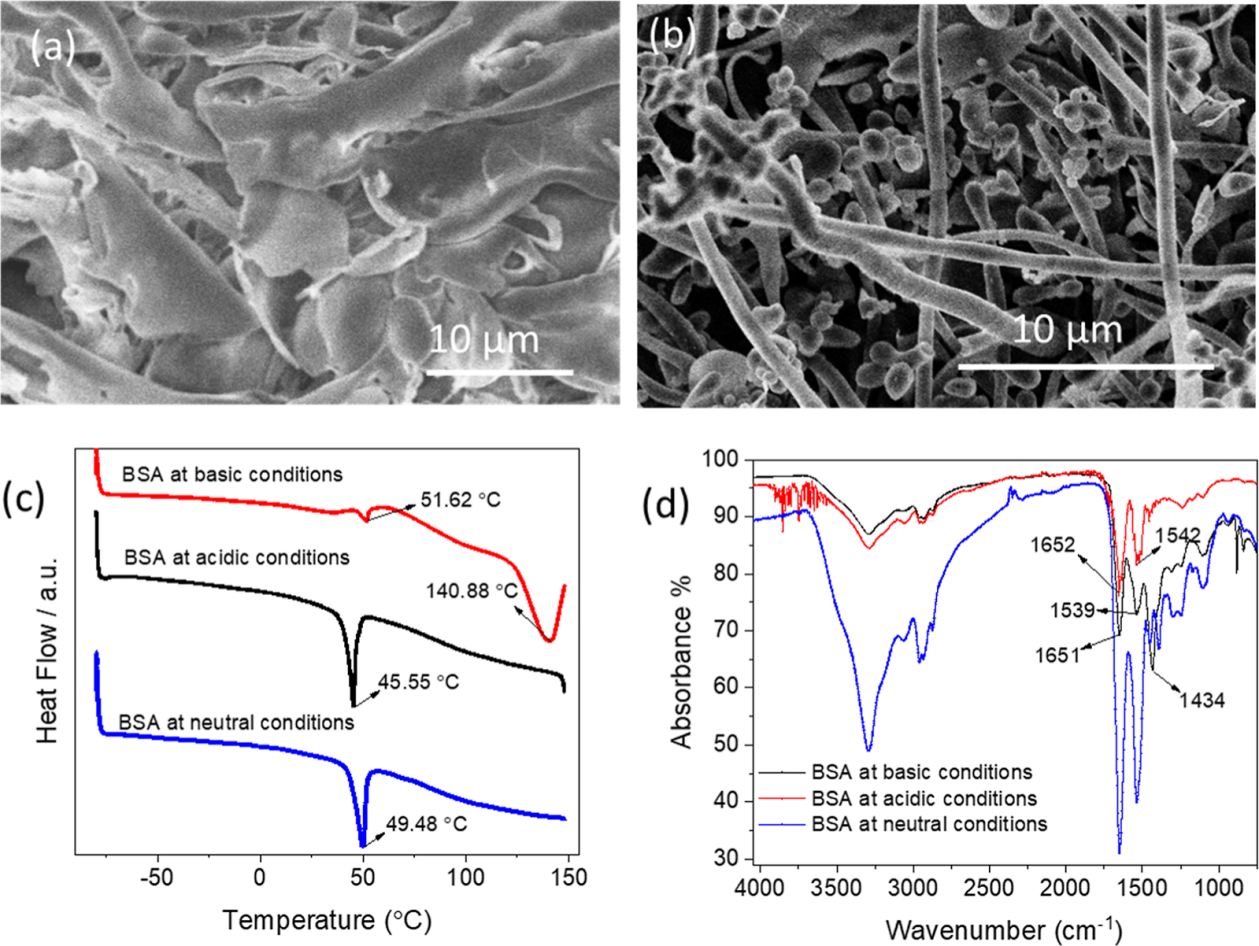
Freeze-dried BSA prepared from 1.5 mg/mL BSA solutions at different pHs. Morphologies of ice-templated BSA from pH 2 (a) and pH 10 (b). (c) DSC profiles and (d) IR spectra of the BSA materials prepared from BSA solutions under acidic (pH = 2), neutral (pH = 7), and basic (pH = 10) conditions.

To provide insights into the formation of microspheres, we further studied the influence of aqueous BSA solution pH on the morphology of ice-templated BSA materials. When the solution pH was reduced to 2, a large sheet-like structure was observed (Figure 2a), while the resulting material showed fibers and microspheres with some aggregates at pH = 10 (Figure 2b). According to the DSC results (Figure 2c), the BSA material prepared from pH 2 presented no glass transition temperature but showed a melting peak at around 45.55 °C, which was lower than that of BSA prepared under neutral conditions (49.48 °C). Indeed, BSA presents different conformations in water depending on the solution pH, that is, fully expanded conformation (E-form) at pH < 2.7; fast conformation (F-form) at pH 2.7–4.3; normal conformation (N-form) at pH 4.3–8; basic conformation (B-form) at pH 8–10; and aged form (A-form) at pH > 10.^31,32^ Unfolding of intradomain helices in domain I and domain III occurs during conformational changes from a compact heat shape (N-form) to a linear expanded conformation (E-form), resulting in the decrease of melting point of BSA under acidic conditions.^33,34^

This was further confirmed by the FT-IR spectra (Figure 2d). Freeze-dried BSA from acidic condition showed a peak absence of amide III vibration at around 1434 cm^–1^ and a narrow band amide II at 1542 cm^–1^ but still displayed amide I vibration at 1652 cm^–1^ (Figures 2d and S5a), indicating the unfolding of domain III and the helical structure of domain I during a N-to-E transition.^35,36^ The shape of E-form BSA is an oblate spheroid with about 35% α-helix content, which is lower than 55% α-helix content of N-form BSA.^37^ It may be unfeasible for E-form BSA molecules to develop fibrous structures or nanospheres during freeze-drying.

With an isoelectronic point at pH 4.7–5.2, B-form BSA is a negatively charged macromolecule, of which protein chains do not fully extend in basic aqueous solution (pH 10).^38^ This difference between BSA under basic and acidic conditions could result in different BSA morphologies, as observed in Figure 2. For the BSA material prepared under the basic condition, as shown in Figures 2d and S5a, the peak at 1434 cm^–1^ demonstrates the presence of amide III vibration and the intensity ratio of amide I/amide II bands at 1651 and 1539 cm^–1^ remains similar to N-form BSA. This indicates a relatively complete folding structure of B-form BSA with an approximate 48% α-helix content.^31,37,39^ Owing to the subtle changes of secondary and tertiary structures in the N–B transition, a similar melting point should have been obtained.^40^ Nevertheless, the BSA material prepared under basic conditions showed two melting peaks at 51.62 and 140.88 °C (Figure 2c), both of which were higher than the melting point of BSA under neutral condition. This could be attributed to the minor amount of sodium hydroxide remaining from the preparation of basic BSA solution.

Based on the above analysis, the solution pH has an impact on the formation of microspheres. However, there is no simple direct link that could be established. It has been noticed that BSA microspheres could be formed after the ice-templating process, while polymeric nanofibers were produced for polymers such as PVA and chitosan.^16,17^ Due to the difference in their molecular structures, it was imperative to know if there were any preformed nanostructures in the BSA solutions that could play a role in the formation of microspheres. Therefore, DLS analysis was performed on BSA solutions with different concentrations.

**Figure 3:**
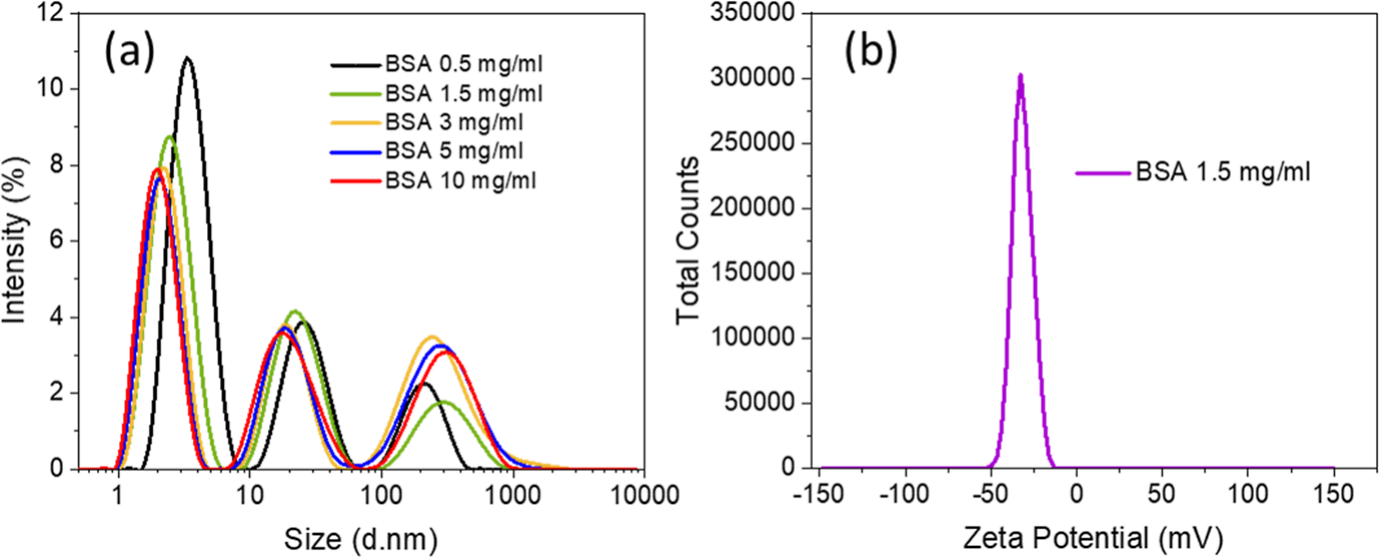
(a) DLS profiles of aqueous BSA solutions at different concentrations. (b) Zeta potential of 1.5 mg/mL BSA solution.

It was found that the BSA solution at the concentration of 0.5 mg/mL showed multiple peaks with the first peak at 3.58 ± 0.02 nm, the second peak at 27.4 ± 0.3 nm, and the third one at 216.4 ± 4.4 nm in order of decreasing percentages of intensity area (Figure 3a and Table S1). The BSA solution of 1.5 mg/mL had a similar distribution of peaks with the first peak at 2.59 ± 0.01 nm, second peak at 24.2 ± 1.1 nm, and third peak at 234.7 ± 2.4 nm. With the increase of BSA concentration to 3 and 5 mg/mL, in contrast, the percentages of intensity area at 325.8 ± 4.6 and 327.2 ± 4.6 nm became higher than that at 20.1 ± 0.4 and 20.5 ± 0.2 nm, respectively. As for BSA at the concentration of 10 mg/mL, the intensity areas percentage of peak 2 (20.9 ± 0.1 nm) and peak 3 (333.7 ± 4.0 nm) were close to each other (Table S1). Moreover, the zeta potential of BSA at concentrations of 1.5 and 5 mg/mL presented negative values in the same order, with −31.74 ± 0.62 and −38.94 ± 0.28 mV (Figures 3b and S5b), respectively.

As a globular protein, BSA exhibits a three-dimensional heart-shaped structure, composed of polar amino acids on the outside and nonpolar amino acids arranged on the inside.^9^ Due to the BSA-water interactions and disrupted hydrogen bond networks of water molecules at the interface of BSA, water molecules are adsorbed on the surface of the BSA molecule, forming a hydration layer.^41^ The hydration layer is regarded as a hydrophilic outer shell while hydrophobic amino acids, including the cross-linked 17 disulfide bridges of cysteine amino acid, constitute the inner core. Most of BSA molecules exist individually in very dilute aqueous solutions.^9^ When the protein concentration increases to a certain value, the hydration layers of BSA molecules overlap and the inner hydrophobic cores are likely to autoassemble, aggregating into larger nanospheres.^42,43^ This is the difference between BSA solutions and aqueous PVA/chitosan solutions where the DLS analysis showed some aggregates across a very wide size range (5–500 nm for 1 mg/mL PVA solution and 100–5000 nm for 1 mg/mL chitosan solution, Figure S6). It is believed that ice nucleation and ice crystal growth occur when BSA solutions are frozen in liquid nitrogen, leading to the isolated aggregation of such BSA nanoparticles into microspheres during the freezing process. This may be analogous to the additive occlusion in CaCO_3_ crystals under suitable crystal growth conditions.^44,45^ Both dyes and charged particles (anionic and cationic) have been embedded in the CaCO_3_ crystals. The crucial requirement is a balance of interactions between additives and crystal surface, i.e., strong enough to allow engulfment of additive particles by the crystal growth but not too strong to inhibit the crystal growth.^44,45^ For the freezing of BSA solution, in order to engulf aggregated BSA nanoparticles to form microspheres, a similar balance may have to be achieved under suitable conditions, that is, the interaction between BSA nanoparticles with the crystals should be sufficiently strong so that BSA nanoparticles are not simply excluded from the freezing front, but not too strong so that BSA nanoparticles are simply engulfed and embedded within ice crystals. This fine balance of interaction shall allow the aggregation of BSA nanoparticles into microspheres that have reduced surface energy and are engulfed by the growing ice crystals.

**Figure 4:**
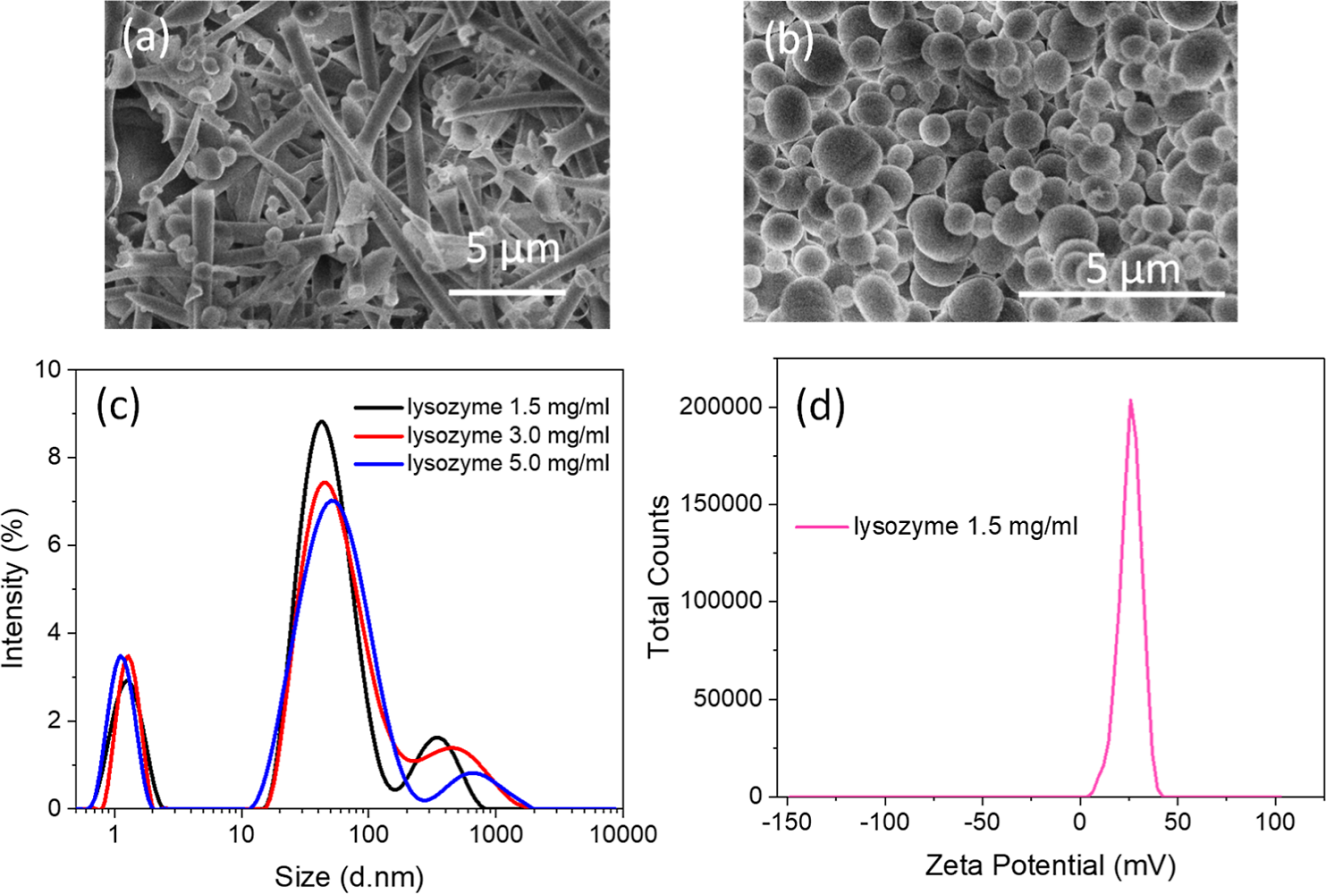
Characterization of freeze-dried lysozyme and lysozyme solutions. (a) Morphology of freeze-dried lysozyme from 1.5 mg/mL solution. (b) Morphology of BSA: lysozyme at a mass ratio of 2:1 at the concentration of 1.5 mg/mL. (c) Size distribution of lysozyme solutions at different concentrations. (d) Zeta potential of 1.5 mg/mL lysozyme solution.

In further effort to explore the formation mechanism and what factors are important for the formation of microspheres, other globular proteins, lysozyme and papain, were also investigated. When lysozyme solutions were freeze-dried, fibrous structures were mainly produced from concentrations of 1.5 mg/mL (Figure 4a). Increasing the concentration to 3.0 mg/mL still gave rise to nice fibrous structures (Figure S7a). However, further increase of the lysozyme concentration to 5 mg/mL produced a material with a small percentage of fibers but more lumps (Figure S7b). It was noticed that there was no sample with majority of microspheres formed from aqueous lysozyme solutions. It was then studied to mix lysozyme solution and BSA solution, trying to establish whether the presence of BSA was crucial for the formation of microspheres by this simple ice-templating method. When the lysozyme solution was blended with BSA solution at the mass ratio of 1:1 and 1:2, the structures were still mainly composed of fibers, with the presence of some microspheres (Figure S7c,d). However, when the ratio of BSA: lysozyme was increased to 2:1, i.e., BSA was the dominating component, BSA-lysozyme composites showed microspheres formed (Figure 4b). The sizes of these microspheres were varied, accompanied by a very small number of long nanofibers. This indicates the important role of BSA in the formation of microspheres.

We further investigated whether this could be attributed to the self-assembly process to form nanospheres in the BSA solutions, as shown in Figure 3a. As such, DLS analysis and zeta potential measurement were performed on aqueous lysozyme solutions. Figure 4c shows the DLS profiles of lysozyme solutions at different concentrations. A triple size distribution, with the peaks around 1 nm, 50 (the main peak), and >200 nm, can be observed. The peak intensities vary with the changing of lysozyme concentration (Table S2). This is consistent with the DLS results of BSA solutions, where with concentration increasing to a certain value, the second largest percentage of peak area intensity moves to hundreds of nanometers and returns to smaller nanometers at higher concentrations. However, the zeta potential of the lysozyme is significantly different from that of BSA solutions. The lysozyme solutions at concentrations of 1.5 and 5 mg/mL showed positive values of 25.80 ± 0.52 mV (Figure 4d) and 37.10 ± 1.27 mV (Figure S8a), while the zeta potential of BSA solutions was negative. Conversely, BSA/lysozyme nanocomposites at a 2:1 mass ratio showed a zeta potential of 0.21 ± 0.06 mV (Figure S8b) and a narrowly monodispersed peak at 9.15 ± 0.21 nm (Figure S8c). Based on the microspheres observed and the zeta potentials of the starting protein solutions in Figures 1–4, it suggests that the surface charge of self-assembled protein nanospheres may be crucial for the formation of microspheres during the ice-templating process. The negative or close to neutral surface charge could be in favor for the formation of microspheres over nanofibers at a suitable protein concentration, although we do not have a theoretical explanation or experimental data to directly support this statement at this stage.

**Figure 5:**
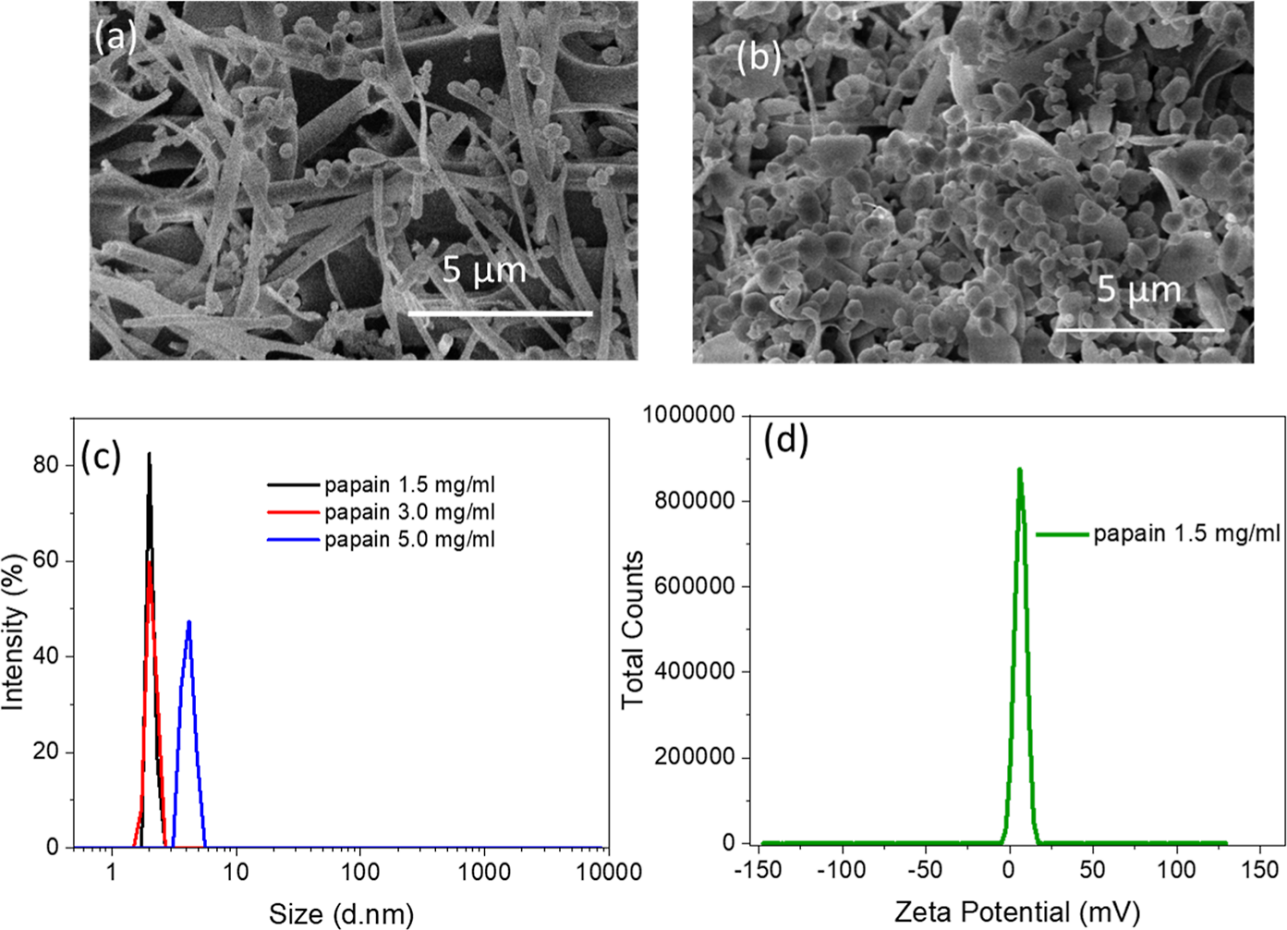
Characterization of freeze-dried papain and papain solutions. (a) Morphology of freeze-dried papain from 3 mg/mL solution. (b) Morphology of BSA: papain at a mass ratio of 2:1 at the concentration of 1.5 mg/mL. (c) Size distribution of papain solutions at different concentrations. (d) Zeta potential of 1.5 mg/mL papain solutions.

This statement was further supported by the materials prepared involving papain. For papain, mainly nanofibrous and small amount of microspherical structures were observed at 1.5 and 3 mg/mL (Figures S9a and 5a). Nevertheless, bigger lumps were formed and the size of the microspheres did not increase when the concentration was increased to 5 mg/mL (Figure S9b). When papain was mixed with BSA at mass ratios of 1:1 and 1:2, BSA-papain composites were composed of large lumps and irregular microspheres (Figure S10a,b). With the mass ratio at 2:1 of BSA and papain, BSA-papain composites were mainly composed of microspheres (Figure 5b), indicating that the participation of BSA was vital for the production of microspheres. According to the DLS analysis, papain at concentrations of 1.5, 3, and 5 mg/mL displayed a narrowly monodispersed peak at 2.1 ± 0.3, 2.1 ± 0.1, and 4.1 ± 0.1 nm (Figure 5c). Moreover, positive values of 6.52 ± 0.34 mV (Figure 5d) and 4.40 ± 0.43 mV (Figure S11) were determined for the papain solutions at both concentrations of 1.5 and 5 mg/mL. When the BSA solution was mixed with papain solution at different mass ratios of BSA/papain (2:1, 1:1, and 1:2), the zeta potential of the mixing solution showed a change from negative value of −3.16 ± 0.23 mV (2:1 in Figure S12a) to positive values of 0.72 ± 0.08 mV (1:1 in Figure S12b) and 1.88 ± 0.08 mV (1:1 in Figure S11c). All of the mixing BSA and papain solutions showed a bimodal size distribution (Figure S12d and Table S3). This observation from the papain system is consistent with what was observed for the BSA and BSA/lysozyme (2:1) study above—the negative surface charge may enhance the formation of microspheres. However, the surface charge of polymer nanoparticles in aqueous solution may not be the sole or main contributing factor. A more systematic study with different types of proteins, nanoparticle size and surface charge, and general hydrophilic polymer systems is required to provide direct evidence for the formation mechanism.

### 3.2 Use of BSA Nanofibers/Microspheres as Scaffolds for the Upload and Release of Curcumin

Considering diverse properties of BSA in terms of biodegradability, biocompatibility, nontoxicity, and nonimmunogenicity, we further evaluated the use of BSA nanofibers/microspheres as scaffolds for loading a model hydrophobic drug curcumin.^46^ We selected the condition where BSA microspheres/nanofibers were formed (1.5 mg/mL aqueous BSA solution frozen in liquid nitrogen) for the drug loading and release study, also taking into account the potential impact of loaded curcumin on scaffold morphology. Nanoprecipitation and soaking evaporation methods were employed for the loading of curcumin.

**Figure 6:**
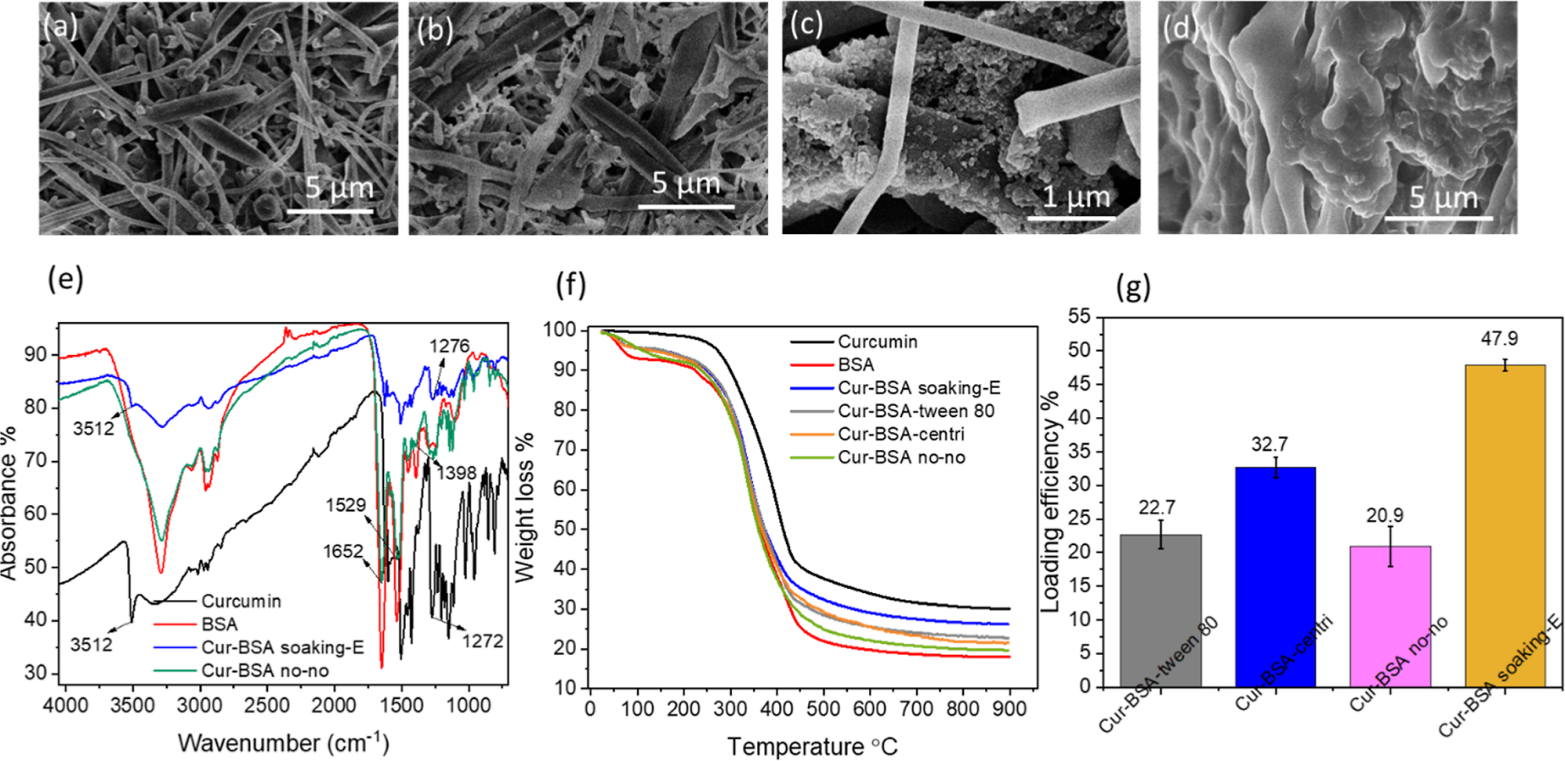
Characterization of Cur-BSA composites. Morphology of Cur-BSA composites prepared by nanoprecipitation with (a) or without Tween 80 (b), without Tween 80 and subsequent centrifugation process (c), and by soaking evaporation method (d). IR spectra (e), TGA profile (f), and drug loading efficiency of Cur-BSA composites (g).

We found that centrifugated curcumin nanoprecipitates (named Cur-BSA-Tween 80) individually distributed in BSA nanofibers/microspheres in a rice-seed-like form, when Tween 80 was added as a surfactant in the nanoprecipitation process (Figure 6a). Without the addition of Tween 80, clusters of rice-seed-like curcumin particles (named Cur-BSA-centri) were scattered in BSA nanofibers/microspheres (Figure 6b). Additionally, rice-seed-like particles and curcumin nanoparticles (named Cur-BSA no–no) were both observed in BSA nanofibers/microspheres without Tween 80 and subsequent centrifugation in the nanoprecipitation process (Figures 6c and S13). The curcumin particles prepared (before further processing with BSA solution) were observed (Figures S14 and S15), demonstrating the successful incorporation of curcumin particles in the BSA scaffolds. When fabricated by the soaking evaporation process, curcumin nanoparticles (named Cur-BSA soaking-E) aggregated together in tighter BSA nanofibers (Figure 6d).

The Cur-BSA composites were further characterized by FTIR. Compared to the spectrum of BSA, the peak attributed to stretching vibration of phenolic O–H in Cur-BSA soaking-E appeared at 3512 cm^–1^, suggesting the existence of curcumin after the soaking evaporation process (Figure 6e). New peaks at 1529 and 1276 cm^–1^ were due to stretching vibration of benzene ring and aromatic C–O of curcumin in Cur-BSA no–no, Cur-BSA-Tween 80, Cur-BSA-centri, and Cur-BSA soaking-E (Figures S16a and 6e).^47^ Similar to the spectrum of curcumin, peaks belonging to C–H aromatic vibration in Cur-BSA-Tween 80 and Cur-BSA-centri showed up at 1033 and 1012 cm^–1^ (Figure S16a).^48^ Additionally, bands related to vibrations of amide III, amide II, and amide I in Cur-BSA no–no, Cur-BSA-Tween 80, Cur-BSA-centri, and Cur-BSA soaking-E were detected at 1398, 1529, and 1652 cm^–1^, indicating that BSA conformation was relatively intact in these composites (Figure 6e).^49,50^

According to TGA in Figure 6f, among the composites, Cur-BSA soaking-E showed the highest residual mass (approximate 26.2%) at 900 °C, followed by Cur-BSA-Tween 80 (about 22.8%) and Cur-BSA-centri (around 21.6%). The lowest residual mass belonging to Cur-BSA no–no was about 19.7%. We found that all residual masses of these samples were between 30.1% (curcumin) and 17.9% (BSA), indicating that different quantities of curcumin were loaded in the drug delivery system. This result is consistent with drug loading efficiency. The highest drug loading efficiency belonged to Cur-BSA soaking-E with 47.9% (Figure 6g). Cur-BSA-centri and Cur-BSA-Tween 80 possessed 32.7 and 22.7% of curcumin loading efficiency, respectively. As expected, these composites displayed elemental contents different from those of BSA and curcumin (Table S4). In contrast with C and N contents of BSA (48.5 and 13.7%), higher C content and less N content were detected for Cur-BSA soaking-E (57.9 and 7.7%), Cur-BSA-centri (55.4 and 9.1%), Cur-BSA-Tween 80 (53.1 and 11.0%), and Cur-BSA no–no (52.7 and 11.1%).

**Figure 7:**
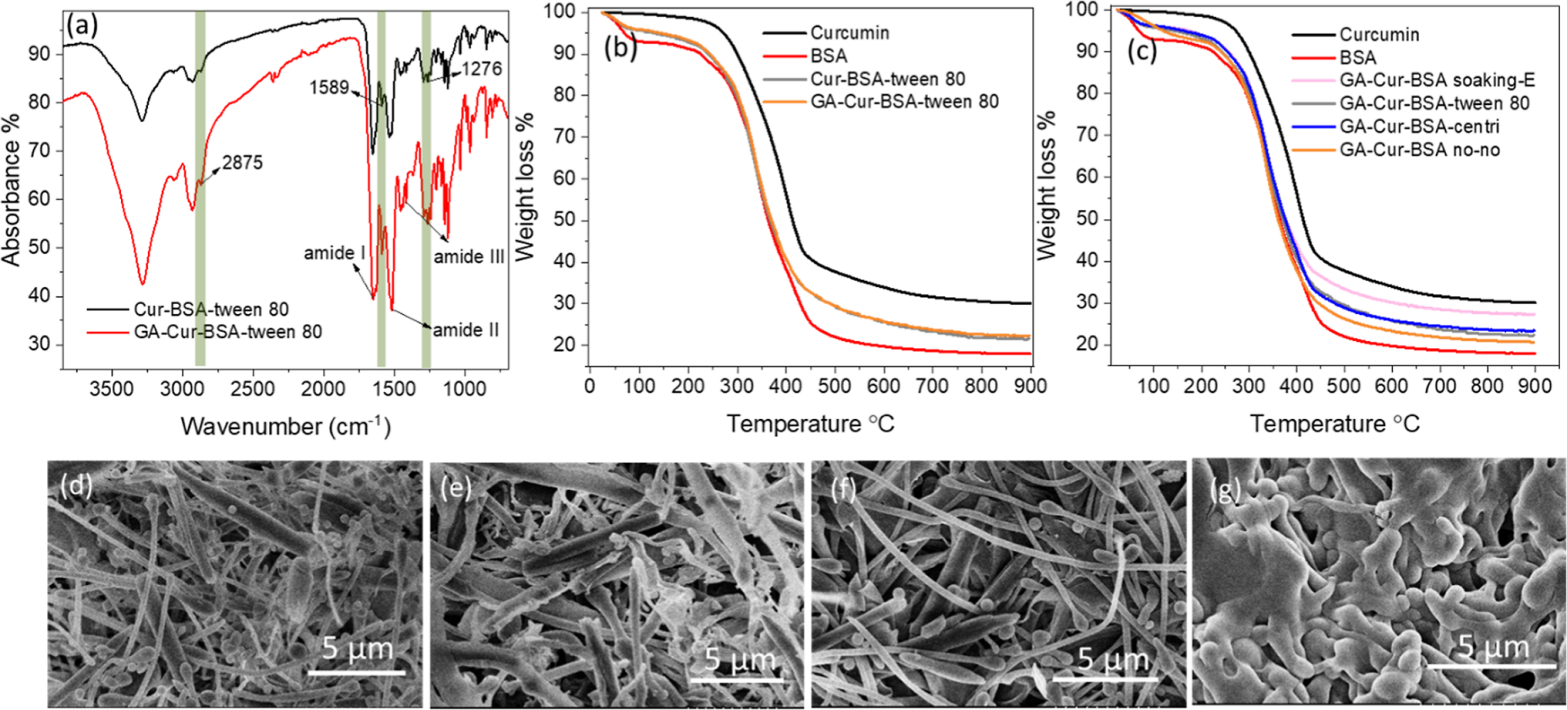
Characterization of Cur-BSA composites after cross-linking by GA vapor. FTIR spectra (a) and TGA profile (b) of Cur-BSA-Tween 80 before and after GA vapor cross-linking. (c) TGA profile of four Cur-BSA nanocomposites treated by GA vapor. Morphology of Cur-BSA nanocomposites after GA vapor treatment prepared with (d) or without Tween 80 (e), without Tween 80 and the subsequent centrifugation process (f), and soaking evaporation method (g).

Due to the high solubility of BSA in water, this drug delivery system could dissolve immediately in aqueous medium, resulting in the burst release of curcumin. To address this issue, GA vapor cross-linking was performed to improve stability of these composites in wet environment. Although GA is toxic, after removing GA after the cross-linking reaction, the GA-cross-linked materials showed noncytotoxicity on human foreskin fibroblasts cells and good cell viability (more than 80%) for human decidua parietalis placental stem cells.^51,52^ FTIR spectra of Cur-BSA-Tween 80, Cur-BSA-centri, Cur-BSA no–no, and Cur-BSA soaking-E treated with GA vapor exhibited peaks for amide III, amide II, and amide I (Figures 7a and S16b–d). Peaks attributed to stretching vibration of benzene ring and aromatic C–O of curcumin still showed up at 1589 and 1276 cm^–1^, suggesting GA vapor cross-linking treatment did not damage curcumin. Due to the presence of aldehydes in both curcumin and GA, stretching vibration of O=C–H was overlapped at 2875 cm^–1^ (Figures 7a and S16b–d).^53^

Moreover, Cur-BSA composites exhibited similar TGA profiles with close residual mass before and after GA vapor cross-linking (Figures 7b and S17a–c), and the ranking of residue mass after treatment was also the same as that before cross-linking (Figure 7c), suggesting no loss of BSA or curcumin in the GA vapor cross-link treatment. Further characterization by SEM presented that the morphologies of GA-Cur-BSA were similar to Cur-BSA, thus GA vapor cross-linking did not change or damage the microstructures of Cur-BSA (Figure 7d–g). The successful GA cross-linking was further confirmed by the CHN analysis. The carbon contents showed increase trends after GA vapor treatment in Table S4, from 57.9 to 60.4% (Cur-BSA soaking-E), from 52.7 to 53.8% (Cur-BSA no–no), from 55.4 to 56.4% (Cur-BSA-centri), and from 53.1 to 53.9% (Cur-BSA-Tween 80). In contrast, nitrogen contents of Cur-BSA exhibited a downward trend after the GA vapor cross-linking process (Table S4).

### 3.3 Curcumin Release Behavior

**Figure 8:**
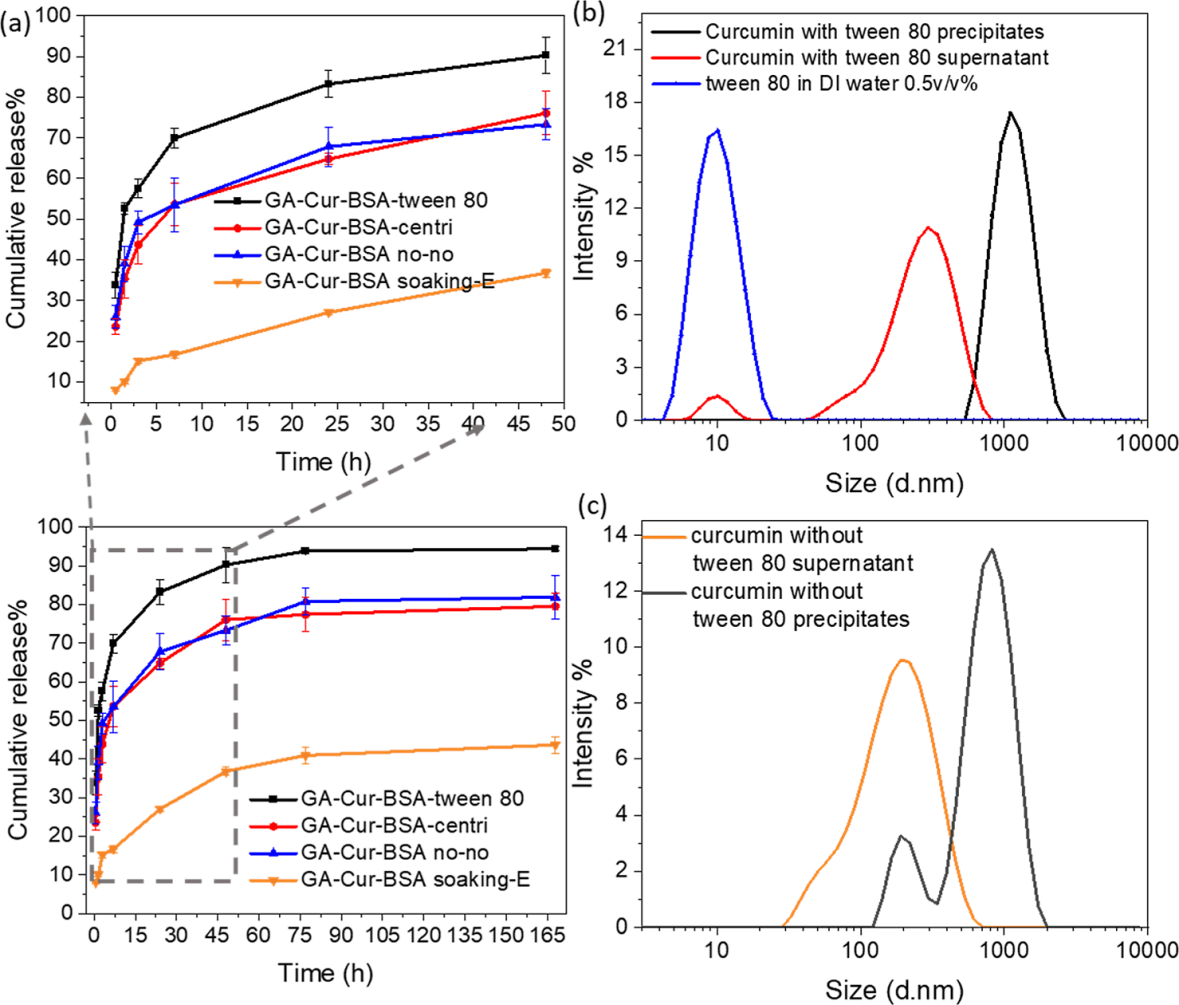
(a) Cumulative release profile of curcumin in ethanol/PBS medium and the expanded region for the release between 0 and 50 h. (b) Size distribution of curcumin prepared by the nanoprecipitation method with and (c) without Tween 80, measured by DLS.

Due to its many excellent properties such as anti-inflammatory, hypoglycaemic, antioxidant, and antimicrobial activities, curcumin has attracted considerable attention.^54^ Despite its high therapeutic efficacy, the low solubility and poor bioavailability of curcumin limits the administration and transport across physiological barriers, which is the general problem among diverse hydrophobic drugs.^55^ The investigation of curcumin release behavior as the model drug could pave the way for other hydrophobic drugs loaded in this protein scaffold for potential treatments of diverse diseases. In alignment with other curcumin-loaded drug systems where PBS was generally mixed with solvents (methanol and ethanol) or surfactants (Tween 80), we chose the same aqueous–organic solvent release medium, i.e., an ethanol–water mixture with volume ratio at 1:1.^56,57^ GA-Cur-BSA-Tween 80 showed the fastest release behavior, reaching a cumulative percentage release of 90.25% at 48 h and 94.38% at 168 h, followed by 76.04 and 79.50% for GA-Cur-BSA-centri, as well as 73.28 and 81.89% for GA-Cur-BSA no–no (Figure 8a). Particularly, the curcumin release from GA-Cur-BSA soaking-E was very slow, with a cumulative release of only 36.76% at 48 h and 43.64% at 168 h. A plateau release was achieved from 48 to 168 h (Figure 8a).

We investigated whether the release differences among these GA-Cur-BSA nanocomposites could be explained by the size of the curcumin particles in the composites. During the preparation of the composites, a curcumin/ethanol solution was injected into DI water in the presence or absence of Tween 80. After centrifugation, the obtained orange precipitates were dispersed in BSA solutions for in situ encapsulation. As measured by the DLS analysis, the precipitates exhibited a monodispersed peak at 1110.5 ± 4.5 nm for curcumin with the presence of surfactant (Figure 8b). The supernatant phase from the sample curcumin-Tween 80, on the contrary, showed a bimodal size distribution with the first peak at 295.0 ± 4.8 nm and the second one at 10.1 ± 0.2 nm. The second peak was attributed to the residues of Tween 80, which was confirmed by the single peak at 10.4 ± 0.063 nm of Tween 80 in DI water (Figure 8b).

As for the supernatant phase from the sample curcumin-without-Tween 80, only the monodispersed peak was determined at 190.2 ± 0.6 nm (Figure 8c). Curcumin precipitates in the absence of Tween 80 showed a bimodal distribution with the first peak at 955.1 ± 3.6 nm and second peak at 190.4 ± 2.3 nm (Figure 8c), indicating that a small amount of curcumin nanoparticles at the size of around 200 nm remained in the precipitates after centrifugation and hence in the subsequently prepared composite. When comparing the release profiles of curcumin (Figure 8a) and on account of similar size distribution of curcumin particles (prepared with and without Tween 80) in the Cur-BSA composites (Figure 8b,c), it was speculated that the surfactant on the curcumin particle surface instead of particle size contributed to the fast release of curcumin. The presence of Tween 80 in the composite GA-Cur-BSA-Tween 80 could lead to better wettability to the curcumin particles and facilitate the dissolution and release of curcumin, hence, the faster release profile.

**Figure 9:**
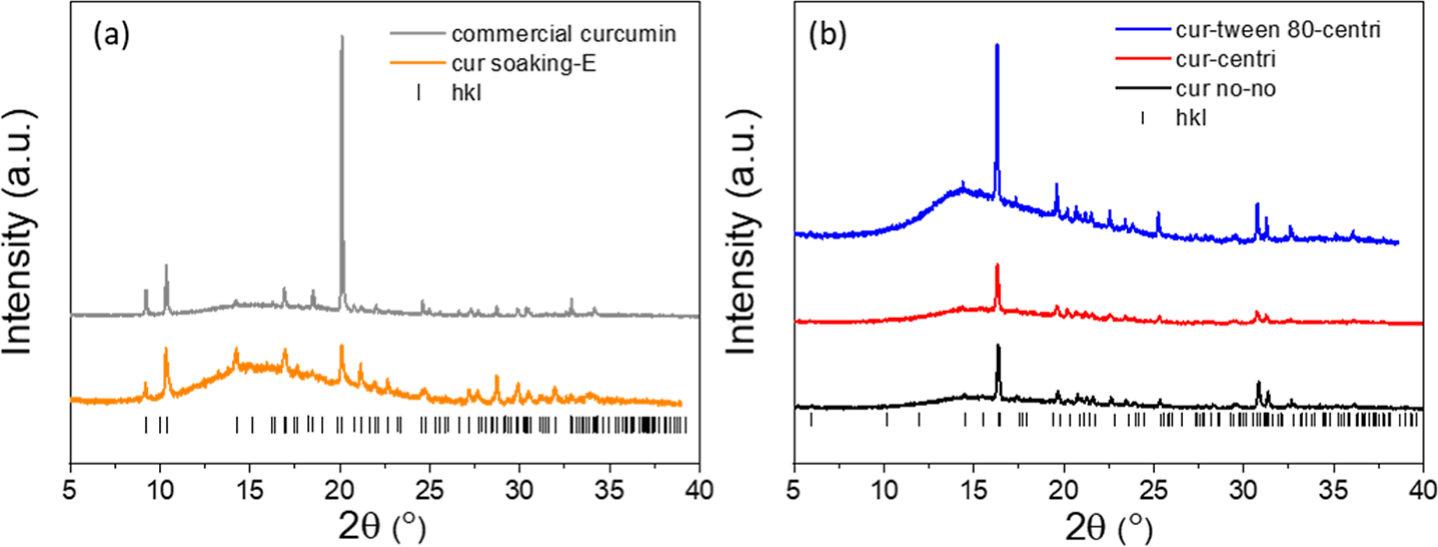
PXRD pattern of curcumin particles produced by different methods: (a) by solution evaporation (cur soaking-E) and the as-purchased curcumin; (b) by nanoprecipitations with Tween 80 and subsequent centrifugation (cur-tween 80-centri), without tween 80 but with centrifugation process (cur-centri), and without either of them (cur no–no).

To further elucidate the release behavior of curcumin, PXRD analysis was performed on the curcumin particles by the same methods but without the involvement of BSA. PXRD patterns of commercial curcumin and the curcumin particles prepared by solution evaporation (Figure 9a) matched with the known Form 1, which is a monoclinic crystal structure and consistent with the findings in literature.^58,59^ On the contrary, as shown in Figure 9b, PXRD patterns of cur-tween 80-centri, cur-centri, and cur no–no, which were prepared by nanoprecipitation with or without surfactant and centrifugation, were indexed to the orthorhombic crystal structure (Form 3).^59,60^ A macrocylic ring is formed by four curcumin molecules via hydrogen bonds between enolic carbonyls and phenols, comprising stable twisted conformation of Form 1.^59^ Instead, Form 3 consists of one crystallographic independent molecule via C–H···O interactions with enolic O–H, constituting planar conformation.^59−61^ These diverse conformations hinder solvent–solute interactions to different extents.

As revealed in the literature,^59^ the solubility of Form 3 is the highest in ethanol–water medium, whereas the solubility of Form 1 is less than half of that of Form 3, which is in good agreement with the release profiles of curcumin discussed above. Specifically, GA-Cur-BSA-Tween 80, GA-Cur-BSA-centri, and GA-Cur-BSA-no-no with curcumin in Form 3 crystal structure displayed higher solubility and fast release behavior in aqueous media. Furthermore, owing to the discrete distribution of curcumin particles and the presence of Tween 80, GA-Cur-BSA-Tween 80 showed a faster release behavior than that of GA-Cur-BSA-centri and GA-Cur-BSA-no-no (Figure 8a). By comparison, GA-Cur-BSA soaking-E with Form 1 curcumin particles showed the lowest release profile due to the lowest solubility. This suggests that the crystalline form of curcumin particles in the composites instead of curcumin loading plays a more important role in the release of curcumin. Therefore, different preparation methods could be utilized to control the crystalline phase and loading of curcumin particles in the composites, hence achieving the controlled release of hydrophobic drug from the protein scaffolds, in contrast with other drug delivery scaffolds.^56,62,63^

We then investigated whether it was possible to release curcumin in PBS without the addition of any organic solvents due to the expected enhanced dissolution rate and solubility of the nanoparticles prepared from poorly water-soluble compounds, which was a result of controlling the crystalline phase, size, and loading of curcumin.^16^ Because of the low solubility of curcumin in water (0.6 μg/mL), the calibration curve of curcumin in PBS could not be obtained.^64^ Without quantifying the amount of curcumin released, the release of curcumin into PBS from different composites was monitored by comparing the UV–vis absorbance of the PBS solutions at 427 nm.^65^ These PBS solutions were taken from the PBS release medium when the same mass of each composite was placed in 30 mL of PBS.^65^ GA-Cur-BSA-Tween 80 showed the fastest release behavior within 24 h (Figure S18a). However, a further gradual release of curcumin from GA-Cur-BSA soaking-E was observed, achieving the highest cumulative release at 72 h. This can be attributed to the highest loading of curcumin in GA-Cur-BSA soaking-E (Figure 6g). GA-Cur-BSA no–no displayed a much lower curcumin release profile within 72 h, followed by that of GA-Cur-BSA-centri. This is believed to be the result of low curcumin loading and a crystalline phase.

The stability of GA-Cur-BSA composites under the releasing conditions in PBS was also evaluated by determining the amount of BSA dissolved from the scaffolds using the Bradford test. Aqueous protein solutions can be normally detected by UV–vis at an absorbance wavelength of 280 nm. However, released curcumin also showed some absorbance around 280 nm. To accurately measure the released BSA, Bradford test was employed for protein quantification in this study. The Bradford reagent can be bound to proteins and shifts the absorbance maximum to 596 nm, which could be easily monitored by UV–vis analysis.

As shown in Figure S18b, the lowest remaining mass percentage (based on BSA scaffold mass) was for GA-Cur-BSA-Tween 80 (73.86%) within 22 h and followed by GA-Cur-BSA soaking-E after at 192 h (68.61%). The significant mass loss within the initial 22 h could be attributed to the dissolution of not fully cross-linked BSA in the composites. For GA-Cur-BSA soaking-E, the highest amount of curcumin after solvent evaporation could form a thick coating and limit the GA vapor cross-linking process. In the GA-Cur-BSA-Tween 80 composite, the presence of Tween 80 (a long chain nonionic surfactant) could hinder the access of GA vapor and subsequent cross-linking. The other two composites showed better stability, with GA-Cur-BSA-centri exhibiting a remaining mass percentage of 91.06% at 192 h, followed by 89.04% for GA-Cur-BSA no–no. All GA-Cur-BSA composites indeed maintained more than 65% of the scaffold mass and a relatively intact scaffold structure after 192 h. Due to good control on morphology, drug loading and crystalline phase, and scaffold stability, these BSA scaffolds prepared by simple ice-templating and post-treatment could lay the foundation for controlled drug release in pharmaceutic and biomedical fields.

## 4 Conclusions

BSA scaffolds with nanofibrous and microspherical structures were prepared by a simple ice-templating method from aqueous BSA solutions. The morphologies of the BSA materials were influenced by the freezing temperature, solution concentrations, and pH conditions. It was particularly exciting to observe the formation of BSA microspheres because there has been no report on the preparation of polymer or ceramic microspheres via the ice-templating of solutions. This was attributed to BSA nanoparticles existing in the solution phase (via self-assembly of BSA molecules) and a fine balance of the interactions between BSA nanoparticles and ice crystals during the freezing process. It was possible to produce microspheres by this ice-templating approach for mixing protein solutions, where BSA was the dominating component. Further study is required to identify the crucial factors and the mechanism for the formation of microspheres, while the current study suggested that negatively charged protein nanoparticles self-assembled in aqueous solutions could facilitate the formation of BSA microspheres.

The ice-templated BSA materials were then used as scaffolds for the controlled release of hydrophobic drugs. Curcumin was loaded on BSA scaffolds by nanoprecipitation and soaking evaporation approaches. In order to improve the stability of the BSA scaffold in an aqueous medium, GA vapor cross-linking was employed to treat BSA/curcumin composites. The resulting BSA/curcumin delivery system presented controlled curcumin release behaviors in PBS/ethanol and PBS media, affected by curcumin loading and the curcumin crystalline phase, with good scaffold stability. It is believed that this protein–drug delivery system can be expanded to load and release a wide range of hydrophobic drugs for treatments of diverse diseases.

## Supporting Information Available

UV–vis calibration curves of curcumin; SEM analysis of freeze-dried BSA, BSA: lysozyme composites, papain, and BSA: papain composites; IR spectra of freeze-dried BSA; DLS profiles of aqueous PVA and chitosan solutions; zeta potential of BSA, lysozyme, and BSA: papain solutions; particle size distribution of BSA solutions, lysozyme solutions, papain, and BSA/papain solutions at different concentrations; SEM graphs of nanoprecipitated curcumin and Cur@ BSA nanocomposites; and FT-IR spectra, TGA, and CHN elemental analysis of Cur@ BSA composites prepared by different methods before and after GA vapor cross-linking (PDF)
