# Supplementary material for: Protein Nanospheres and Nanofibers Prepared by Ice-Templating for the Controlled Release of Hydrophobic Drugs
Source: ACS Appl Nano Mater. 2024 Sep 13;7(18):21692–704. doi: 10.1021/acsanm.4c03657 (PMC11443487; doi:10.1021/acsanm.4c03657)
Supplement: Supplementary file 1 — an4c03657_si_001.pdf [file an4c03657_si_001.pdf]

Supporting Information

Protein Nanospheres and Nanofibers Prepared by Ice-Templating for Controlled Release of Hydrophobic Drugs

Meina Zhang, Hong Cai and Haifei Zhang*

Department of Chemistry, University of Liverpool, Crown Street, Liverpool L69 7ZD, UK

Corresponding author email: zhanghf@liverpool.ac.uk

Supplementary figures: Figures S1 – S18

Supplementary tables: Tables S1 – S4

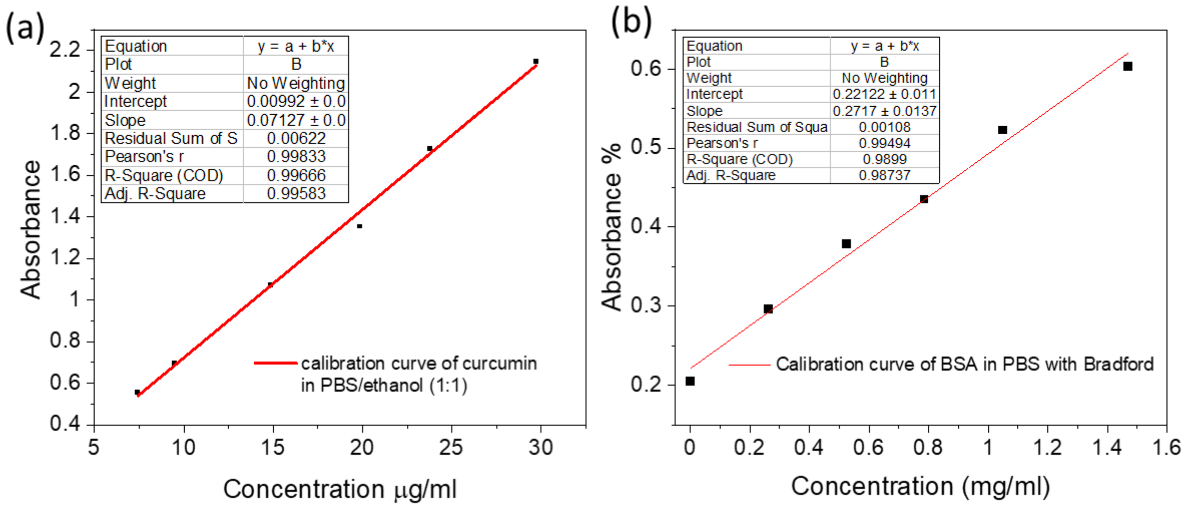

Figure S1. UV-vis **c**alibration curves of (a) curcumin in PBS/ethanol (1:1) based on absorbance wavelength at 432 nm and (b) BSA with Bradford in PBS based on absorbance wavelength at 596 nm.

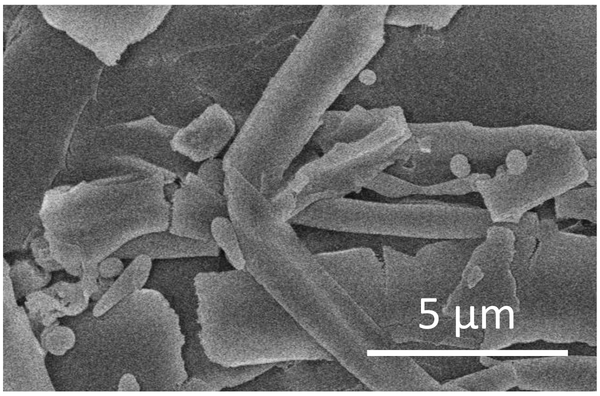

Figure S2. Morphology of the freeze-dried BSA when the BSA solution with the concentration of 10 mg/ml was frozen in liquid nitrogen.

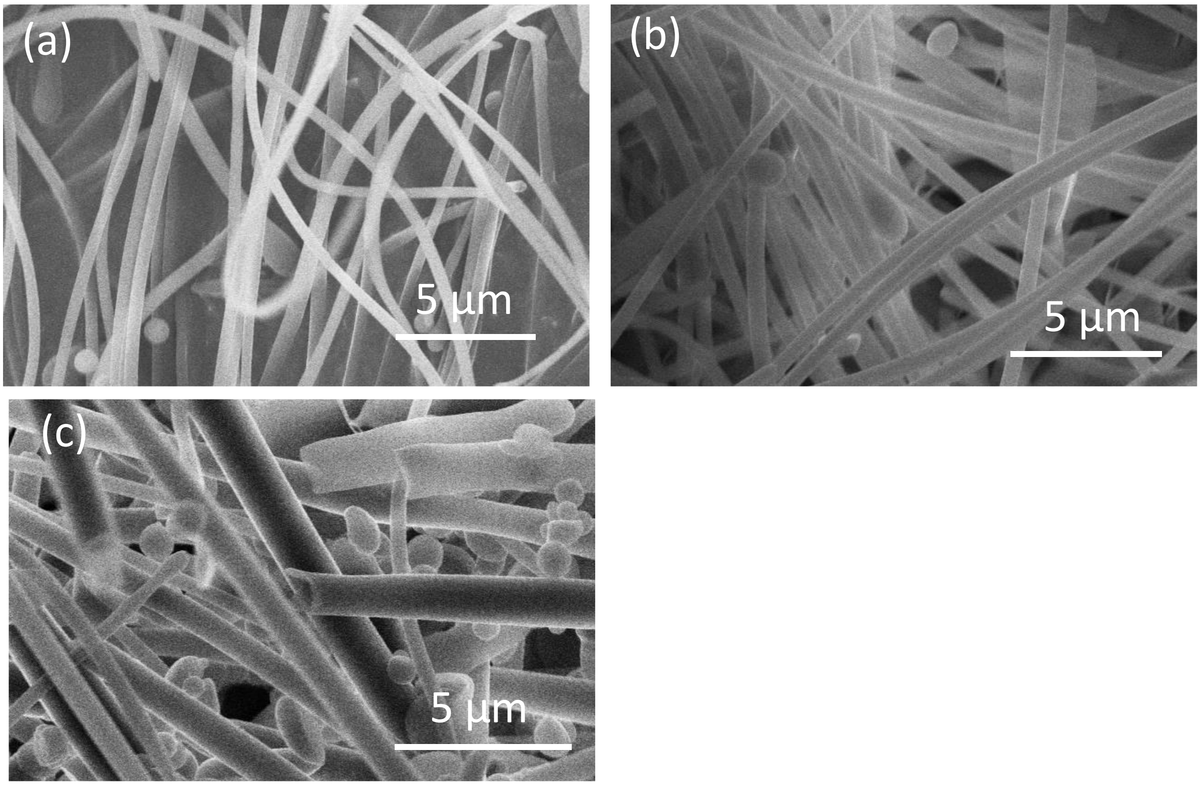

Figure S3. Morphology of the freeze-dried BSA when the BSA solutions with the concentration of (a) 0.5 mg/ml, (b) 1.5 mg/ml, and (c) 3.0 mg/ml were frozen in dry ice/acetone bath (-78 °C).

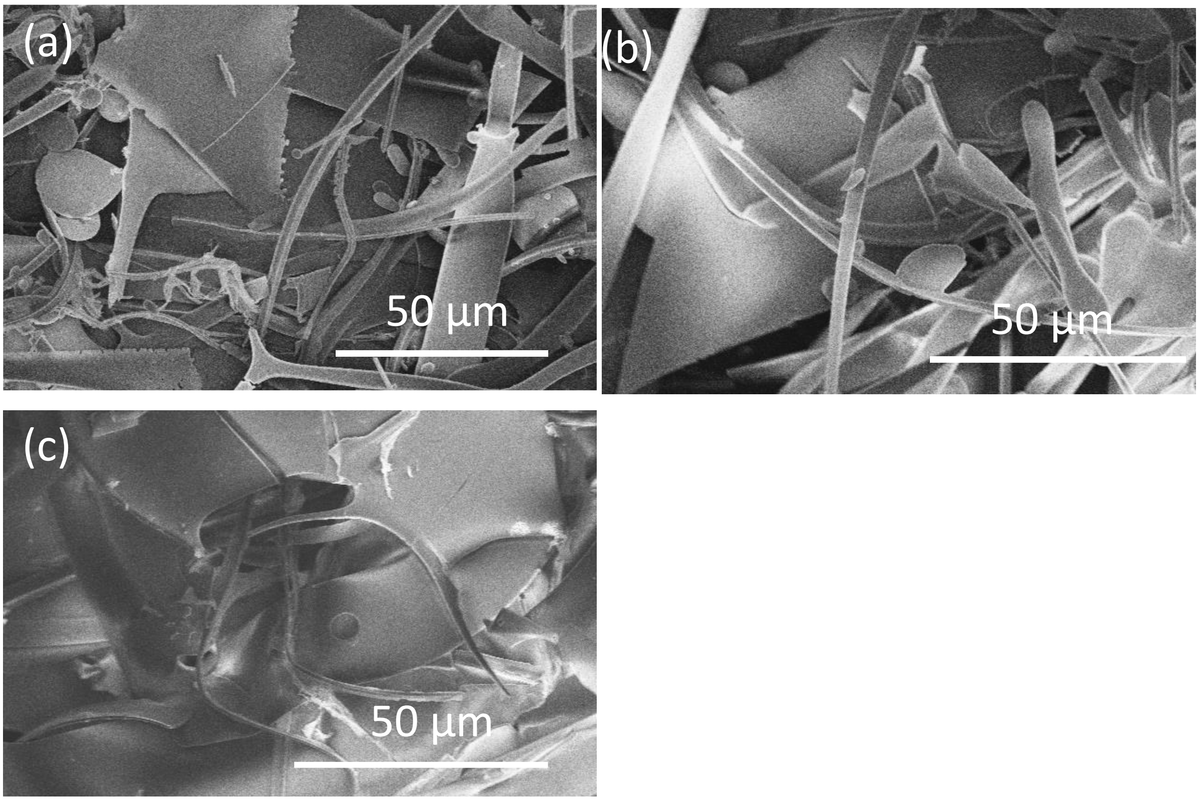

Figure S4. Morphology of the freeze-dried BSA when the BSA solutions with the concentration of (a) 0.5 mg/ml, (b) 1.5 mg/ml, and (c) 3.0 mg/ml were frozen in freezer (-20 °C).

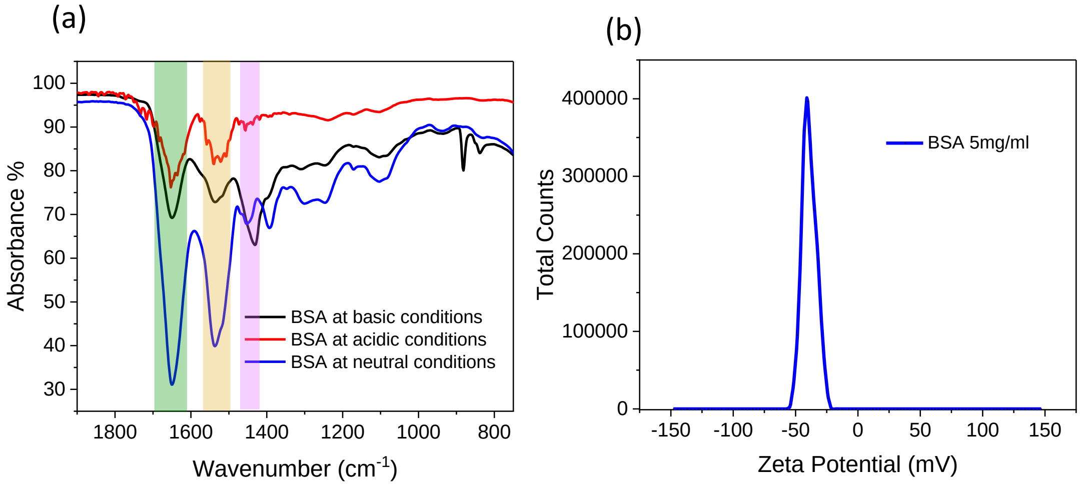

Figure S5. (a) FTIR spectra in the enlarged region of 1800 – 800 cm^{-1} of freeze-dried BSA from the BSA solutions (1.5 mg/ml) at different pH (2, 7, 10). (b) Zeta potential profile of aqueous BSA solution at 5 mg/ml (pH = 7).

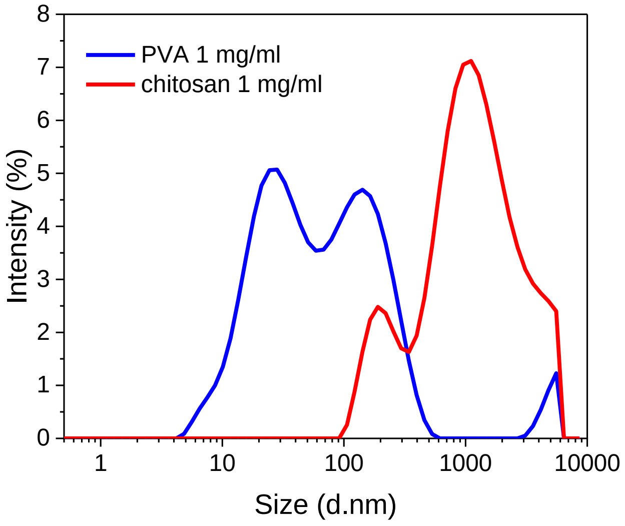

Figure S6. DLS profiles of aqueous PVA solution (molecular weight 89 – 98 kDa, 99+% hydrolyzed, 1 mg/ml) and chitosan (medium molecular weight) solution (1 mg/ml).

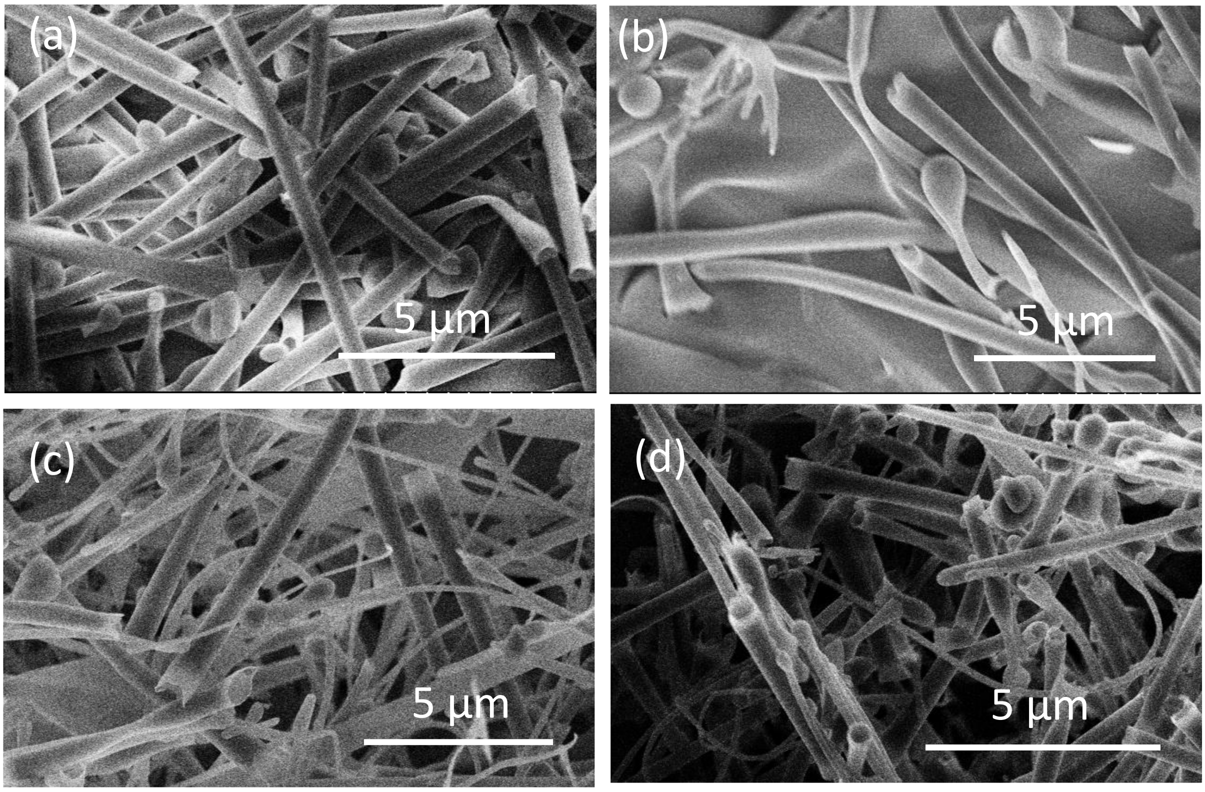

Figure S7. Morphology of freeze-dried lysozyme and BSA: lysozyme composites. (a) prepared from 3 mg/ml lysozyme solution; (b) prepared from 5 mg/ml lysozyme solution; (c) prepared from the BSA: lysozyme = 1:1 at the concentration of 1.5 mg/ml; (d) prepared from the BSA: lysozyme = 1:2 at the concentration of 1.5 mg/ml

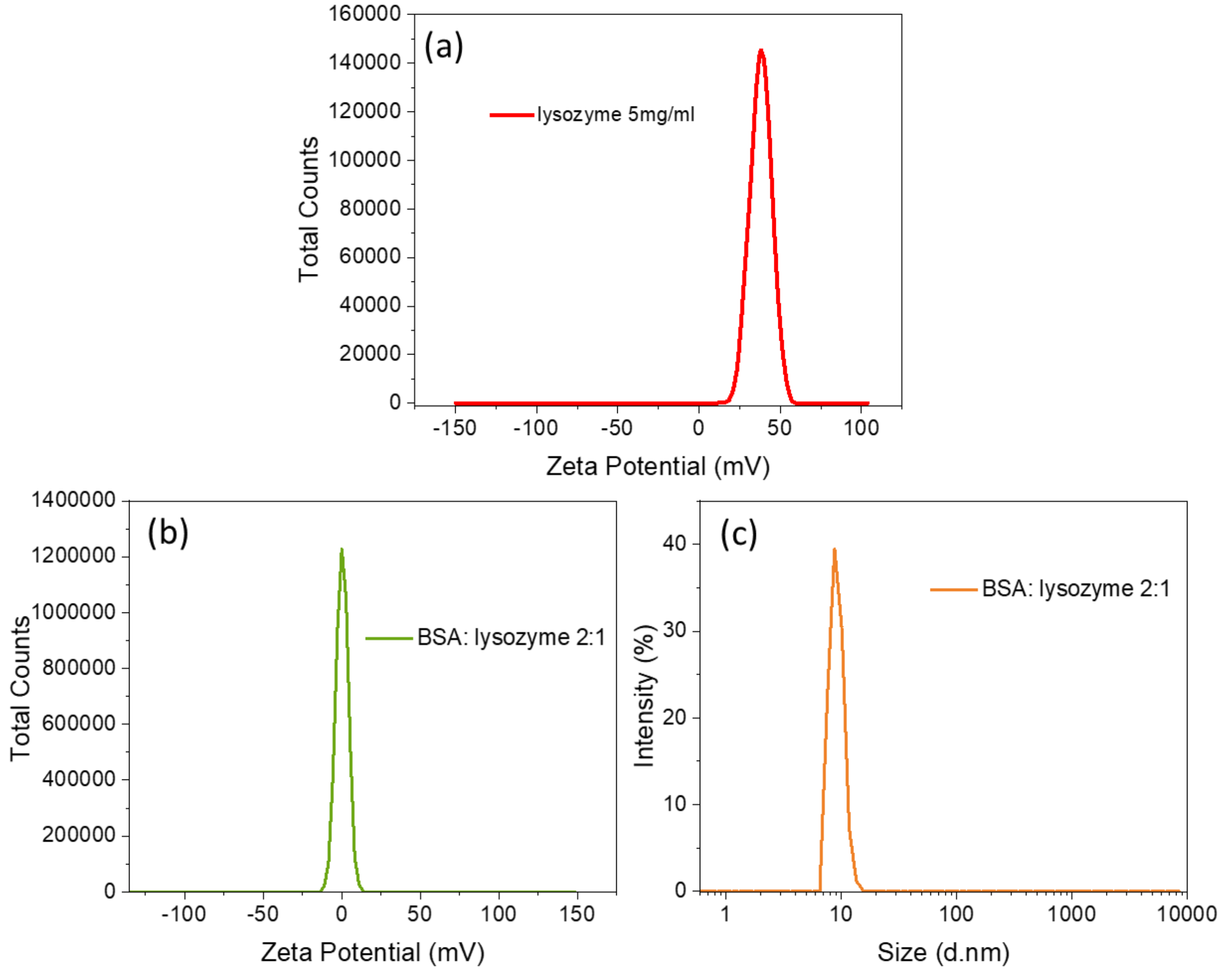

Figure S8. Zeta potential of aqueous lysozyme solution at the concentration of 5 mg/ml (a). Zeta potential (b) and size distribution (c) of BSA: lysozyme at mass ratio of 2:1 at the concentration 1.5 mg/ml.

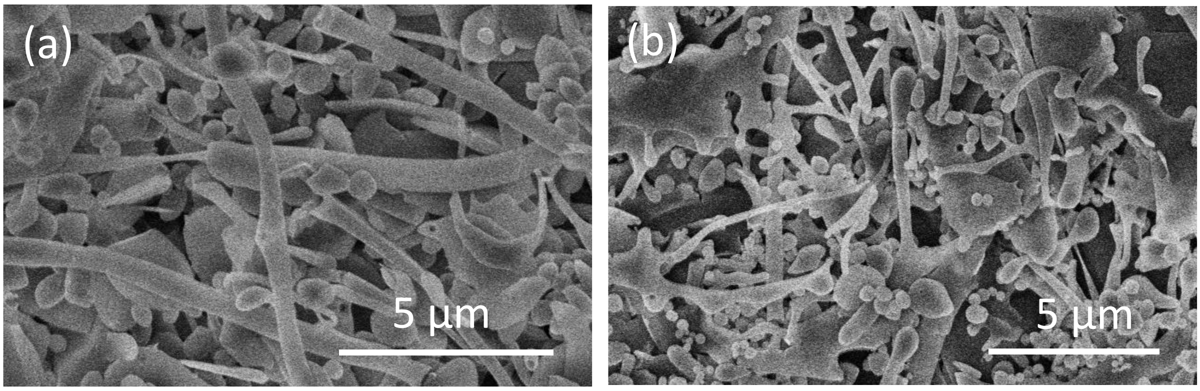

Figure S9. Morphology of freeze-dried papain prepared from (a) 1.5 mg/ml and (b) 5 mg/ml aqueous papain solutions.

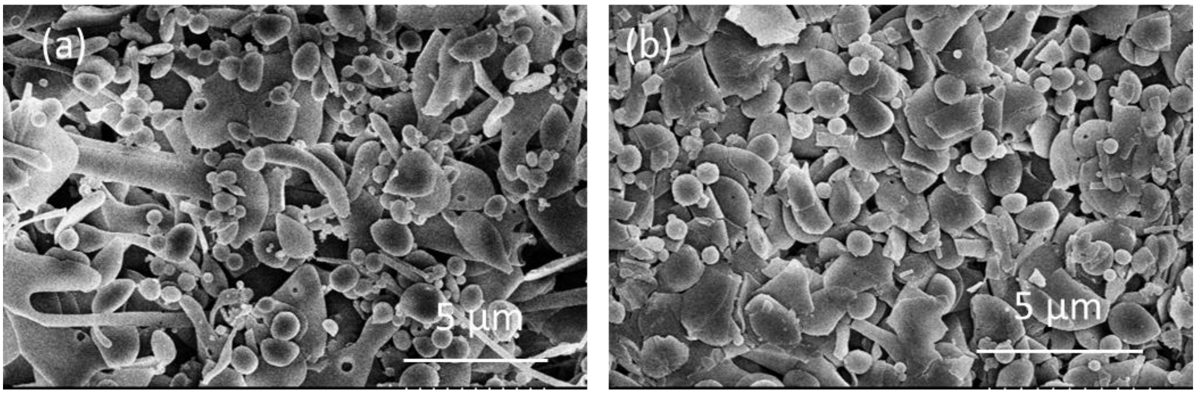

Figure S10. Morphology of freeze-dried BSA: lysozyme composites prepared from (a) aqueous solution with the BSA: papain = 1:1 at the concentration of 1.5 mg/ml; (b) aqueous solution with the BSA: papain = 1:2 at the concentration of 1.5 mg/ml.

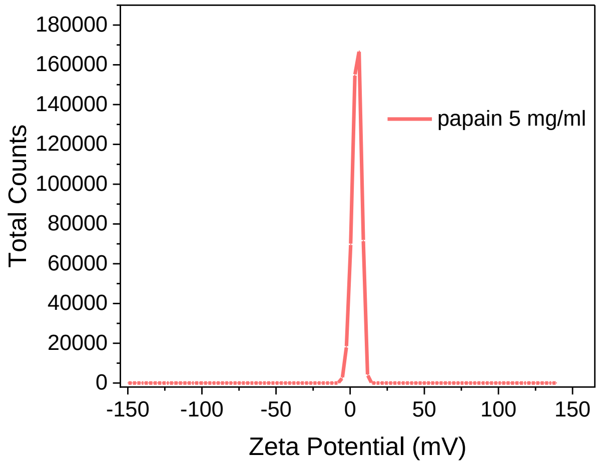

Figure S11. Zeta potential of aqueous papain solution at the concentration of 5 mg/ml.

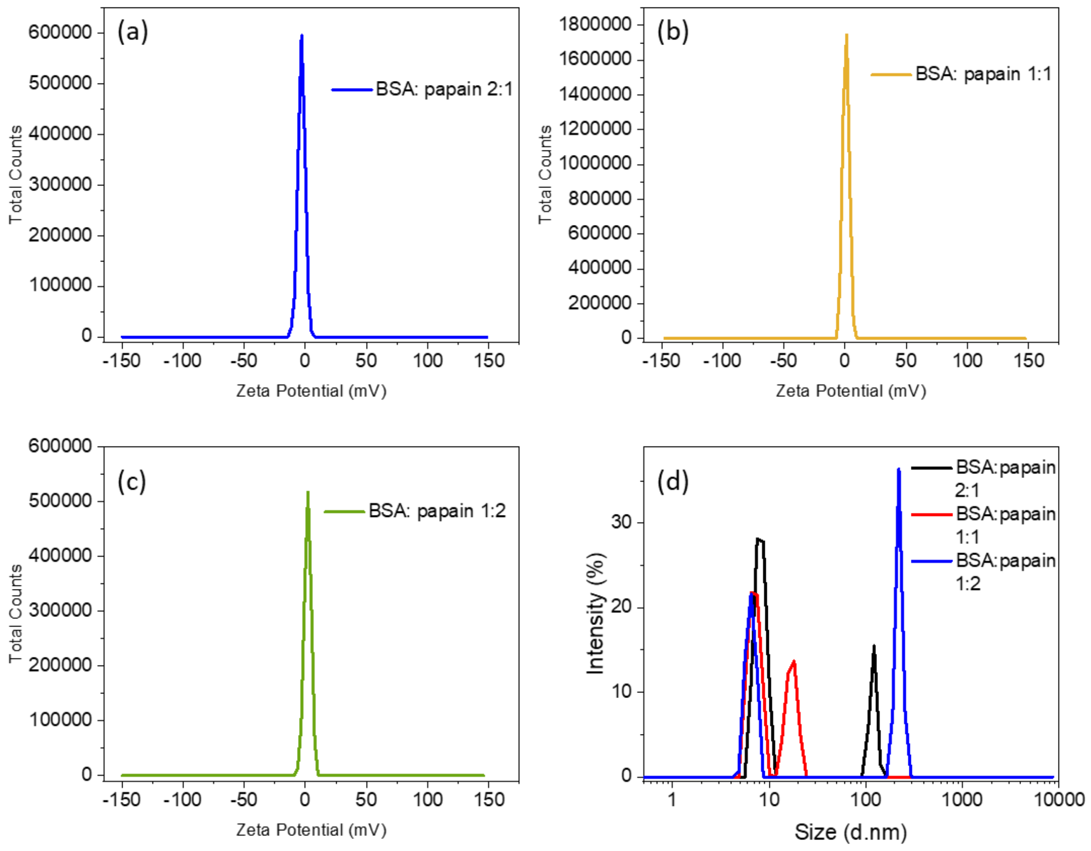

Figure S12. Zeta potential of BSA/papain solutions at the mass ratio of (a) 2:1, (b) 1:1, (c) 1:2 and (d) their particle size at the concentration of 1.5 mg/ml.

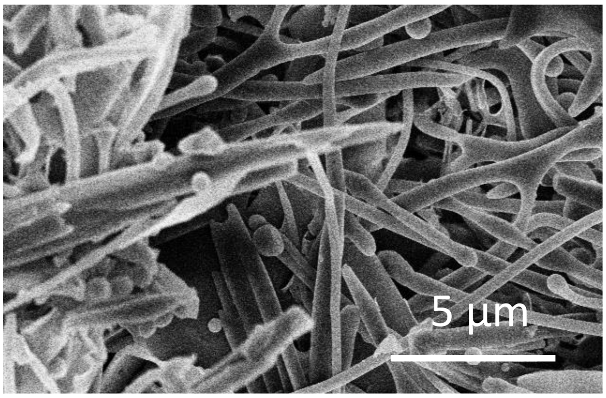

Figure S13. Morphology of Cur-BSA nanocomposites produced without tween 80 and centrifugation process.

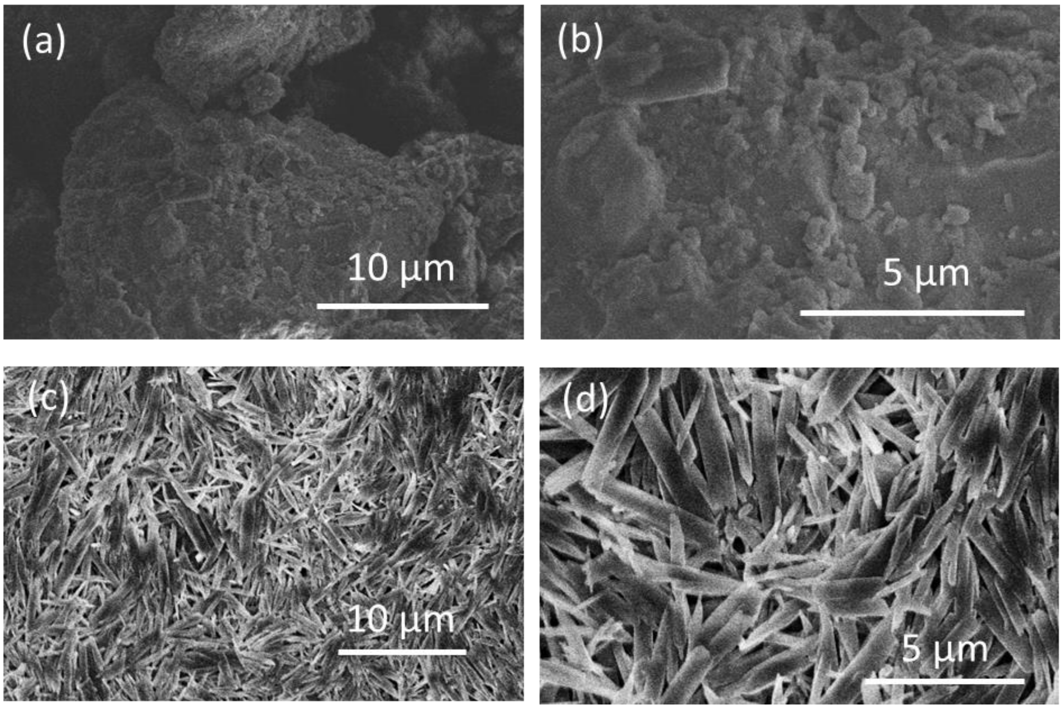

Figure S14. Morphology of as-purchased curcumin (a) and (b), and curcumin particles prepared by nanoprecipitation with Tween 80 and centrifugation (cur-tween 80-centri) (c) and (d) at different magnifications.

.

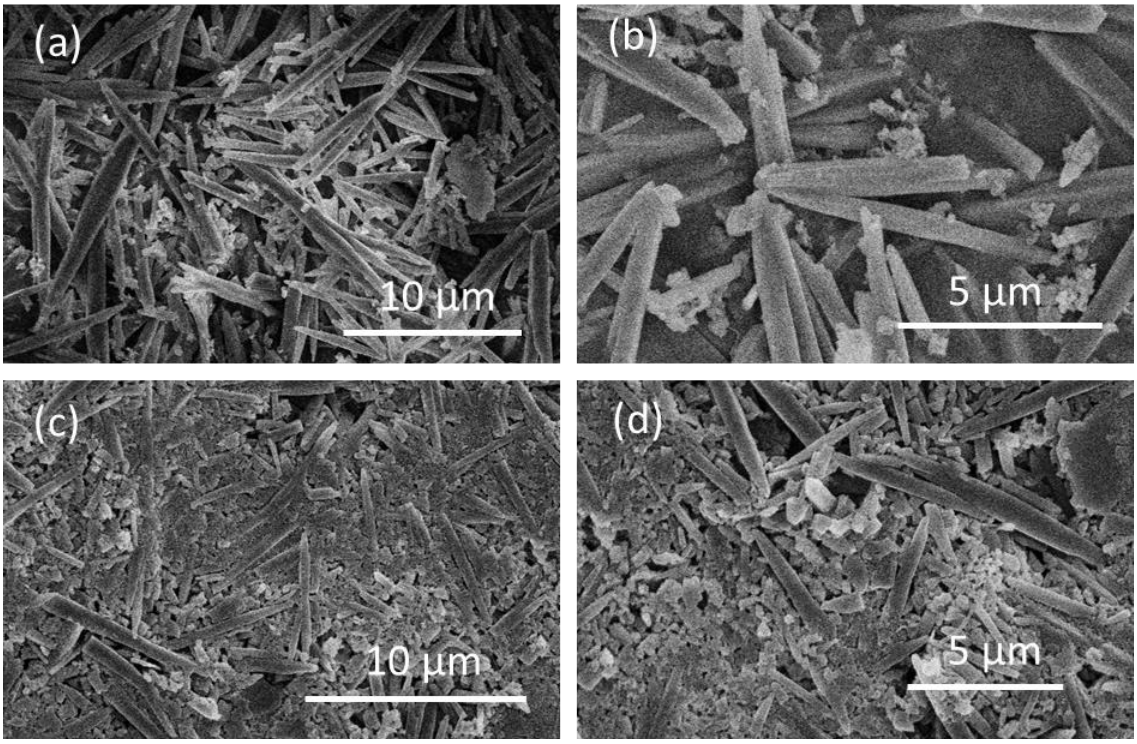

Figure S15. Morphology of curcumin particles prepared by nanoprecipitation without Tween 80 but with centrifugation (cur-centri) (a) and (b) and curcumin particles prepared by nanoprecipitation without Tween 80 and centrifugation (cur-no-no) (c) and (d) at different magnifications.

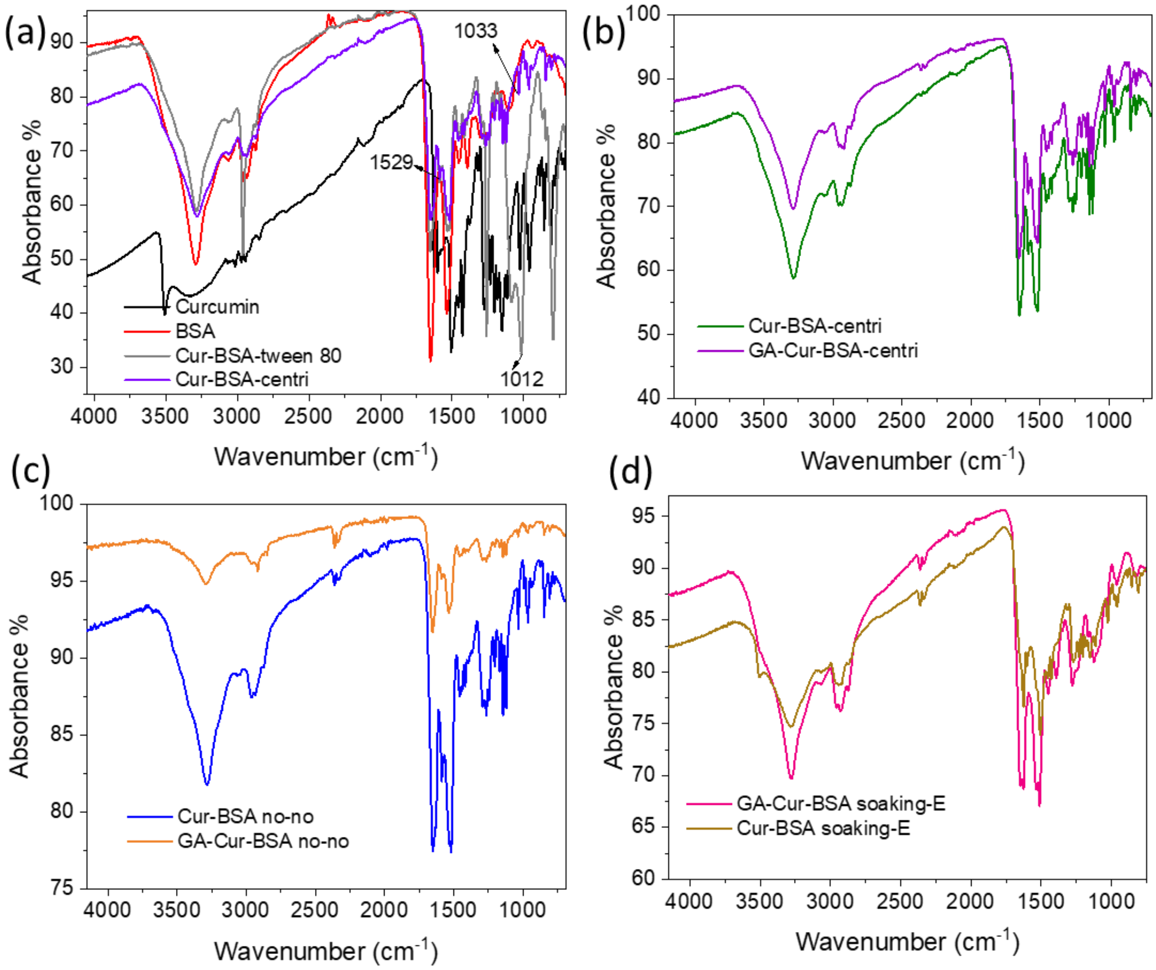

Figure S16. (a) FTIR spectra of Cur-BSA composites, curcumin and BSA. (b), (c) and (d) FTIR spectra of cur-BSA composites prepared by different methods before and after GA vapor crosslinking.

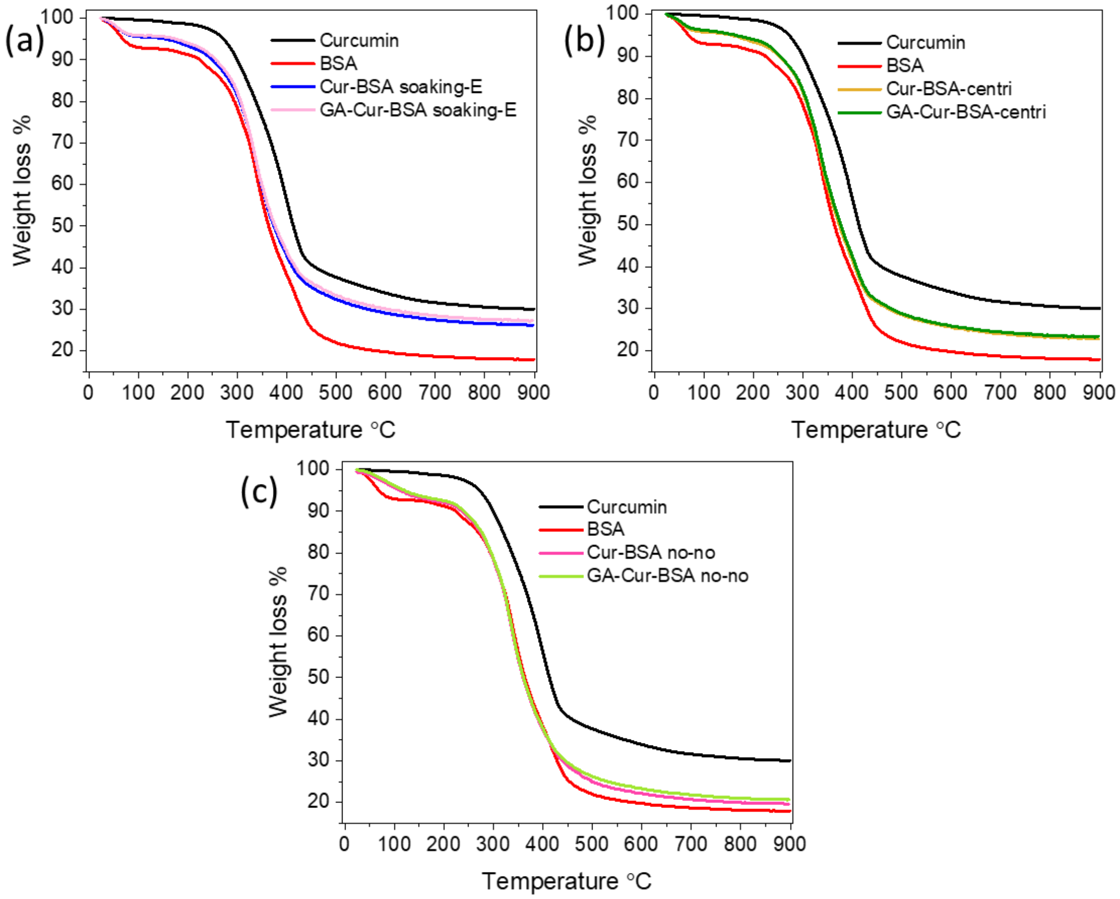

Figure S17. TGA profiles of (a) Cur-BSA soaking-E, (b) Cur-BSA-centri, (c) Cur-BSA no-no before and after GA vapor crosslinking.

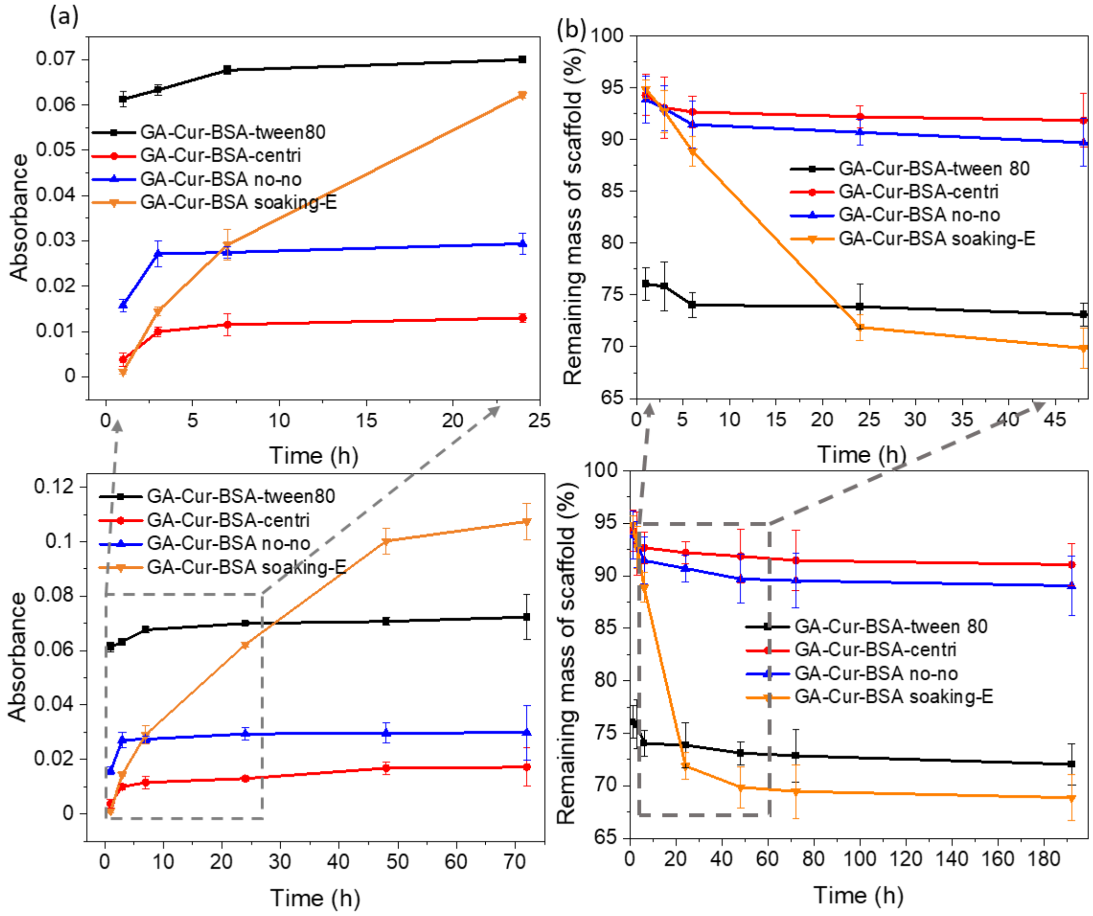

Figure S18. (a) Cumulative release profile of BSA in PBS and the enlarged region for the release at 0 - 25h. (b) The stability of GA-Cur-BSA composites in PBS with time and the enlarged region of 0 - 50 h.

Table S1. Nanoparticle size distribution of aqueous BSA solutions at different concentrations measured by DLS.

	Pk 1 Mean Int (d.nm)	Pk 2 Mean Int (d.nm)	Pk 3 Mean Int (d.nm)	Pk 1 Area Int %	Pk 2 Area Int %	Pk 3 Area Int %
BSA 0.5 mg/ml	3.6 ± 0.1	27.4 ± 0.3	216.4 ± 4.4	62.9	23.9	13.2
BSA 1.5 mg/ml	2.6 ± 0.1	24.2 ± 1.1	234.7 ± 2.4	58.1	28.1	13.8
BSA 3.0 mg/ml	2.3 ± 0.1	20.1 ± 0.4	325.8 ± 4.6	44.6	22.9	32.5
BSA 5.0 mg/ml	2.16 ± 0.1	20.5± 0.2	327.2 ± 4.6	44.4	24.6	31
BSA 10 mg/ml	2.1 ± 0.1	20.9 ± 0.1	333.7 ± 4.0	46.5	27.6	25.9

Table S2. Nanoparticle size distribution of aqueous lysozyme solutions at different concentrations measured by DLS.

	Pk 1 Mean Int (d.nm)	Pk 2 Mean Int (d.nm)	Pk 3 Mean Int (d.nm)	Pk 1 Area Int %	Pk 2 Area Int %	Pk 3 Area Int %
lysozyme 1.5 mg/ml	1.3 ± 0.1	51.1 ± 0.7	354.6 ± 0.3	13.8	75.6	10.6
lysozyme 3.0 mg/ml	1.29 ± 0.1	63.7 ± 1.0	526.8 ± 2.5	11.6	75.2	13.2
lysozyme 5.0 mg/ml	1.16 ± 0.1	61.1 ± 0.6	759.0 ± 5.2	13.6	79.8	6.6

Table S3. Nanoparticle size distribution of aqueous papain and BSA/papain solutions at different concentrations measured by DLS.

	Pk 1 Mean Int (d.nm)	Pk 2 Mean Int (d.nm)	Pk 3 Mean Int (d.nm)	Pk 1 Area Int %	Pk 2 Area Int %	Pk 3 Area Int %
papain 1.5 mg/ml	2.1 ± 0.3	0.0	0.0	100.0	0.0	0.0
Papain 3 mg/ml	2.1 ± 0.11	0.0	0.0	100.0	0.0	0.0
Papain 5 mg/ml	4.1 ± 0.1	0.0	0.0	100.0	0.0	0.0
BSA:papain 2:1	8.2 ± 0.2	219.4 ± 0.2	0.0	77.4	22.6	0.0
BSA:papain 1:1	7.1 ± 0.3	17.2 ± 1.2	0.0	64.6	35.4	0.0
BSA:papain 1:2	6.5 ± 0.1	220.9 ± 5.8	0.0	48.6	51.4	0.0

Table S4. CHN results of Cur-BSA before and after GA vapor crosslinking.

	C	H	N
Curcumin	69.2%	5.6%	0.0%
BSA	48.5%	6.9%	13.7%
Cur-BSA-tween 80	53.1%	6.7%	10.9%
GA-Cur-BSA-tween 80	53.9%	6.6%	10.4%
Cur-BSA -centri	55.4%	6.6%	9.1%
GA-Cur-BSA -centri	56.4%	6.4%	9.0%
Cur-BSA no-no	52.7%	6.5%	11.1%
GA-Cur-BSA no-no	53.8%	6.5%	10.7%
Cur-BSA soaking-E	57.9%	6.4%	7.7%
GA-Cur-BSA soaking-E	60.4%	7.9%	6.9%
